# Fatty Acids/Tetraphenylethylene Conjugates: Hybrid AIEgens for the Preparation of Peptide-Based Supramolecular Gels

**DOI:** 10.3389/fchem.2022.927563

**Published:** 2022-08-08

**Authors:** Elisa Impresari, Alberto Bossi, Edoardo Mario Lumina, Marco Aldo Ortenzi, Josine Marie Kothuis, Graziella Cappelletti, Daniela Maggioni, Michael S. Christodoulou, Raffaella Bucci, Sara Pellegrino

**Affiliations:** ^1^ DISFARM, Dipartimento Di Scienze Farmaceutiche, Sezione Chimica Generale e Organica “A. Marchesini”, Università degli Studi di Milano, Milan, Italy; ^2^ Istituto di Scienze e Tecnologie Chimiche “G.Natta”, Consiglio Nazionale delle Ricerche (CNR-SCITEC), Milan, Italy; ^3^ SmartMatLab Center, Milan, Italy; ^4^ CRC Materiali Polimerici “LaMPo”, Dipartimento di Chimica, Università degli Studi di Milano, Milan, Italy; ^5^ Department of Biosciences, Università degli Studi di Milano, Milan, Italy; ^6^ Dipartimento di Chimica, Università degli Studi di Milano, Milan, Italy; ^7^ Departiment of Food, Environmental and Nutritional Sciences (DeFENS), Università degli Studi di Milano, Milan, Italy

**Keywords:** aggregation-induced emission (AIE), peptide materials, luminescent materials, self-assembly, supramolecular gel

## Abstract

Aggregation-induced emissive materials are gaining particular attention in the last decades due to their wide application in different fields, from optical devices to biomedicine. In this work, compounds having these kinds of properties, composed of tetraphenylethylene scaffold combined with fatty acids of different lengths, were synthesized and characterized. These molecules were found able to self-assemble into different supramolecular emissive structures depending on the chemical composition and water content. Furthermore, they were used as *N*-*terminus* capping agents in the development of peptide-based materials. The functionalization of a 5-mer laminin-derived peptide led to the obtainment of luminescent fibrillary materials that were not cytotoxic and were able to form supramolecular gels in aqueous environment.

## 1 Introduction

Luminescent materials are employed nowadays for technological applications in different fields, such as light-emitting devices, OLEDs, chemical sensing, and bio-imaging (Zhu et al., 2017; Liu Y. et al., 2018; Huang et al., 2020; Meng et al., 2020; Zeng et al., 2020; Zhou et al., 2020; Kalva et al., 2021; Chen et al., 2022; Niu et al., 2022). Fluorescent gels combine the elastic and mechanic features of gels with the additional property of being light emitting, paving the way to advanced applications. As an example, an injectable, photoluminescent hydrogel for the delivery of stem cells into heart and skeletal muscle tissues (Niu et al., 2019) has been recently developed. In this context, supramolecular fluorescent gels are gaining particular interest nowadays as they can address some of the major drawbacks of polymeric ones, e.g., difficult synthesis, process scalability, fluorescence emission, and toxicity (Li et al., 2019). The use of small-molecule emissive gelators has thus been started to be exploited in different applications, as biomedicine (Lim et al., 2019; Rajasekar and Lavanya, 2022) and photoelectronics (Cheng et al., 2020; Cao et al., 2021).

Commonly, organic fluorescent materials are built with conventional π conjugated chromophores, such as fluorescein and rhodamine, that in the solid-state, as well as in concentrated solutions, could form strong π–π stacking interactions. Such aggregates, upon photoexcitation, tend to decay via non-radiative pathways resulting in strong luminescence quenching, often named “aggregation-caused quenching” (ACQ) (Thomas et al., 2007; Huang et al., 2019). This phenomenon practically hampers the use of many chromophores, where intermolecular interaction may occur such as in sensing materials, thus limiting their practical use (Yuan et al., 2010). In 2001, Luo et al. observed that, in some materials, the aggregation plays a constructive role instead of a destructive one in the fluorescence process and coined the term “aggregation-induced emission” (AIE) for this phenomenon (Luo et al., 2001; Hong et al., 2009). Unlike conventional ACQ dyes, AIE luminogens (AIEgens) show negligible or extremely weak emission in dilute solution, while they brightly emit in the solid or aggregate state. Nowadays, numerous AIEgens have been synthesized and widely investigated due to their intrinsic tendency to aggregate and turn on their emission (Martínez-Ávila et al., 2009; Zhang et al., 2014; Arribat et al., 2019; Xu et al., 2020; Meti et al., 2021; Gialelis et al., 2022). AIEgens could indeed be used despite their amount, preventing the use of sophisticated instruments thanks to their high signal-to-noise ratios. Furthermore, the formation of a few emissive aggregates can be readily visualized, improving sensitivity and speed in applications (Liu et al., 2010; Kwok et al., 2015). Tetraphenylethylene (TPE) is the prototype AIEgens due to its easy synthesis and a wide range of possibilities to modify the scaffold (Feng H.T. et al., 2018; Liu Q et al., 2018; Yang et al., 2018; García-González et al., 2021; Ma et al., 2021; Saraswathi and Joseph, 2022). Although some debate still exists, both restriction of intramolecular rotation and intramolecular π–π stacking interactions have been generally recognized as the mechanism of the TPE intense bluish emission (Chen et al., 2019). Substituents present on the TPE phenyl rings can affect its emission properties (Ceballos et al., 2017). It has been reported that para-substituents exert a strong influence on the TPE emission (Yang et al., 2018), as examples, diethylamino TPE shows a yellow emission, while a pyrene derivative emits a white light (Feng X. et al., 2018). On the other hand, the introduction of self-assembling moieties can influence TPE aggregation ability into ordered and well-defined morphologies leading to a wide variety of micro- and nano-architectures (Salimimarand et al., 2017; Talloj et al., 2020; Zhang et al., 2022).

In this work, we developed hybrid compounds combining TPE and fatty acids (FAs). FAs are amphiphilic molecules able to self-assemble into aggregates of various shapes and sizes (Kindt et al., 2020; Singh et al., 2021; Vicente-García and Colomer, 2021). Fluorescent FA derivatives have been found in several applications as direct probes for membranes and liposomes, in the preparation of fluorescent phospholipids, and as biosensors (Muroski et al., 2017; Tokunaga et al., 2017; Gu et al., 2018; Laguerre and Schultz, 2018). Their combination with TPE could lead to hybrid compounds combining the AIE features of TPE and the surfactant properties of FAs. Here, starting from amino-TPE, the functionalization with FAs of different lengths was carried out through amide coupling. The emission properties and the self-assembly tendencies of the obtained compounds were studied by fluorescence, DLS, and electron microscopy. Finally, we investigated the exploitation of the head carboxylic group, for the conjugation of biomolecules such as peptides. In particular, the obtained compounds were used as N-*terminus* capping agents in the development of peptide-based materials. Peptides are indeed able to organize themselves into ordered structures and are widely exploited in the development of smart materials (Clerici et al., 2016; Fuertes et al., 2017; Gagni et al., 2019; Banerjee and Hamley, 2020; Locarno et al., 2020; Vaghi et al., 2020; Varanko et al., 2020; Bucci et al., 2021a; Bucci et al., 2021b; Surís-Valls et al., 2022) and supramolecular gels (Makam and Gazit, 2018; Jain and Roy, 2020; Tsutsumi et al., 2021; Kurbasic et al., 2022). Ultra-short peptides derived from natural proteins, such as amyloidogenic ones, have been investigated, the phenylalanine dipeptide being the most exploited motif (Diaferia et al., 2019; Bucci et al., 2020; Gil et al., 2020; Ji et al., 2021; Criado-Gonzalez et al., 2022). Here, we selected the IKVAV sequence derived from laminin glycoprotein, one of the major components of the basement membrane (Patel et al., 2019), to be functionalized with the developed FA/TPEs conjugates. The laminin 5-mer peptide, once conjugated to highly hydrophobic moieties, is indeed able to induce the amyloid-like fibril formation and it has been used for the preparation of polymeric hydrogels and in the design of bioactive scaffolds (Sahab Negah et al., 2018; Jain and Roy, 2019; Firipis et al., 2021; Yin et al., 2021). Its functionalization with FA/TPEs led to luminescent morphologically different architectures that have been able to form supramolecular gels in an aqueous environment.

## 2 Materials and Methods

The solvents were purchased from common commercial sources and used without additional purification. Zinc, titanium (IV) tetrachloride, succinic anhydride, tetradecanedioic acid, TFA, Dulbecco’s modified Eagle’s medium (DMEM)—low glucose, 2-propanol, Triton X-100, 3-(4,5-dimethylthiazol-2-yl)-2,5-diphenyl tetrazolium bromide (MTT), non-essential amino acids, hydrochloric acid (HCl) and TIPS were purchased from Sigma-Aldrich (Taufkirchen, Germany). Benzophenone was purchased from Merck KGaA (Darmstadt, Germany). 4-Aminobenzophenone, EDC hydrochloride, all the Fmoc-amino acids, HBTU, and HATU were purchased from Fluorochem (Hadfield, United Kingdom). Rink amide AM resin (100–200 mesh, 0.7 mmol/g) was purchased from Novabiochem (Läufelfingen, Switzerland). DIEA was purchased from Iris Biotech GmbH (Marktredwitz, Germany). Dimethyl sulfoxide (DMSO) was purchased from PanReac AppliChem ITW Reagents (Darmstadt, Germany). Penicillin/streptomycin, L-glutamine, and fetal bovine serum were purchased from EuroClone (Pero, Italy).

The peptides were prepared by solid-phase peptide synthesis (SPPS) using the Fmoc/t-Bu strategy on Rink Amide AM resin (100–200 mesh, 0.7 mmol/g). The two peptides were manually synthesized using as coupling systems HBTU/DIEA for the natural amino acids and HATU/DIEA for the two fluorescent hybrid compounds. Once removed from the resin, compounds **4** and **5** were characterized by ESI-MS and NMR techniques and analyzed by RP-HPLC (see Table 1 and SI for the experimental procedure).

**TABLE 1:** Sequence and chemical characteristics of compounds **4** and **5**.

Compound	Sequence	RP-HPLC purity (%)	R_t_	m/z cld.	m/z fnd.
**4**	**1**-IKVAV-NH_2_	99.4	11.4 min^a^	957.56	957.66
**5**	**2**-IKVAV-NH_2_	99.1	13.9 min^b^	1097.71	1098.64

a Gradient elution for compound **4**: 40%–100% of B in A over 20 min at a flowrate of 0.8 ml/min. A: 0.1% TFA in 100% H_2_O; B: 0.1% TFA in 100% CH_3_CN.

b Gradient elution for compound **5**: 50%–100% of B in A over 20 min at a flowrate of 0.8 ml/min. A: 0.1% TFA in 100% H_2_O; B: 0.1% TFA in 100% CH_3_CN.

NMR spectroscopic experiments were carried out either on a 400 MHz Bruker Advance Neo spectrometer (400 and 100 MHz for ^1^H and ^13^C, respectively) or a 500 MHz Bruker BioSpin GmbH (500 and 125 MHz for ^1^H and ^13^C, respectively). Chemical shifts are given in ppm relative to the DMSO-*d*_*6*_ internal standard, and coupling constants (*J*) are reported in hertz (Hz).

Mass spectra were acquired on a Fison MD800 spectrometer and electrospray ion trap on a Finnigan LCQ Fleet spectrometer from Thermo Scientific.

The purity of compounds **4** and **5** was analyzed by analytical RP-HPLC (Jasco LC-NetII/ADC series, equipped with an RP-HPLC Pump PU-4180 and a PDA Detector MD-4010), using a Gemini-NX C18 column from Phenomenex (5 μm, 150 × 4.6 mm). The solvent system used was 0.1% TFA in 100% H_2_O (A) and 0.1% TFA in 100% CH_3_CN (B), the flow rate 0.8 ml/min, column at 30°C, λ 220 nm.

FT-IR spectroscopy measurements were made on a Perkin Elmer Spotlight 400 FT-IR spectrophotometer equipped with a diamond crystal attenuated total reflectance (ATR) accessory. All the sample were analyzed at room temperature in the solid state. A total of 32 scans were performed for all measurements with a resolution of 4 cm^−1^ in the 4,000–650 cm^−1^ spectral region.

CD spectra were measured on a Jasco J-820 spectropolarimeter with a 0.1 cm quartz cuvette. The spectra were recorded from 190 to 250 nm with a 0.2 nm step and a 2 s collection time per step, taking four averages and using a sensitivity of 100 mdeg and a scanning speed of 50 nm/min. The spectrum of the solvent was subtracted to eliminate interference from cell, solvent, and optical equipment. The CD spectra were plotted as the mean residue ellipticity θ (degree*cm^2^*dmol^−1^) versus wavelength λ (nm). Noise-reduction was obtained using a Fourier-transform filter program. The stock solutions of peptides **4** and **5** were prepared in TFE (1 mM, 1 ml) and diluted to 50 µM with different TFE/water mixtures in order to analyze the following conditions: A) 100% TFE, B) 7.5:2.5, C) 1:1, D) 3:7, E) 2:8, F) 1:9, and G) 0.5:9.5 TFE/water mixtures.

TEM samples were prepared by solvent evaporation on a holey carbon grid sample and were observed under a transmission electron microscope L120C (ThermoFisher, United States) operating at 120 KV. Images were acquired by a Ceta camera 4kx4k.

SEM samples were prepared by solvent evaporation on a silicon wafer, both at room temperature and at 60°C, while the gels were transferred as such. All the samples were sputter-coated with gold for 10 s at 0.016 mA Ar plasma (Scancoat six sputter coater) for SEM imaging using an SEM-EDS JSM-IT500 LV (JEOL Spa) operating at high vacuum, which provided the direct visualization of the self-assembled aggregated structures.

For the gel formation, 6 mg of compound **4** or **5** was dissolved in 500 μL of acetonitrile, using alternate ultrasonic bath and vortex until a clear solution was obtained. Then, for compound **4**, 500 μL of a 0.1 M NaHPO_4_ aqueous buffer (pH = 8.4) was added to the solution. After having obtained a clear solution with the aid of an ultrasonic bath and vortex, the sample was left to self-assemble in gel at r.t. overnight. On the contrary, the solution of compound **5** in acetonitrile was diluted with 500 μL of 0.1% (v/v) TFA in water, alternating ultrasonic bath and vortex until a clear solution was obtained. Then, the sample was heated at 65°C for 45 min and cooled down to r.t. for 3 min.

The confocal images were acquired on a Nikon spinning disk confocal microscope, equipped with the CSI-W1 confocal scanner unit. The samples were excited with the 405 nm laser.

UV–vis absorption spectra were obtained on a Shimadzu UV–vis–NIR 3600 spectrophotometer in a 1 cm path length quartz cell. Photoluminescence quantum yields were measured with a C11347 Quantaurus-QY absolute photoluminescence quantum yield spectrometer (Hamamatsu Photonics), equipped with a 150 W xenon lamp, an integrating sphere, and a multi-channel detector steady-state spectrofluorometer (Edinburg Instrument Ltd.). Continuous excitation for the steady-state measurements was provided by a 450 W xenon arc lamp, and the excitation wavelength was set at 325 nm. Photoluminescence lifetime measurements were determined by the time-correlated single-photon counting (TCSPC) method, and they were performed using an Edinburg Pulsed Diode PLED-375 (Edinburg Instrument Ltd.), with a central wavelength of 375 nm. Photoluminescence experiments at room temperature were carried out in various solvent solutions at several concentrations, in the range of 1–2x10^–5^ mol/L in dichloromethane (DCM), dimethylsulfoxide (DMSO)/water mixtures. In all experiments, 10–20 µL of a mother solution 0.26 mM of trifluoroacetic acid (TFA) in DMSO was added to avoid carboxylic acid dissociation or amine-ammonium equilibrium. The acid did not influence the luminescence of the aggregates but rather speed up the aggregation and stabilizes the aggregates.

Lifetime was fitted with a multiexponential decay curves, and the average lifetime was calculated according to $\tau_{\mathrm{av}}\frac{\sum_{n=1}^{m}\alpha_{n}\tau_{n}^{2}}{\sum_{n=1}^{m}\alpha_{n}\tau_{n}}$ **,** *m* is the *nth* component of the fitted decay; *a* is the pre-exponential value of the *nth* component.

For PL measurements, a trace amount of trifluoroacetic acid was added to the measured solution to avoid carboxylic acid dissociation or amine-ammonium equilibrium.

The dynamic light scattering (DLS) measurements were performed using a Malvern Zetasizer Nano instrument (Malvern Panalytical, Ltd.) at 25°C, equipped with a 633 nm solid-state He–Ne laser at a scattering angle of 173°. Analyses were carried out on 50 μM samples dispersed in DMSO/water mixtures by using viscosity and refractive index data reported in LeBel and Goring (1962) after the samples were aged for 24–48 h at rt. The size measurements were averaged from at least three repeated measurements.

Rheological tests were conducted at 25°C using an Anton Paar MCR 302 Rheometer (Anton Paar GmbH), operating in a 25 mm parallel geometry with a 1 mm distance gap. The frequency sweep measurements were performed at 0.8% strain from 0.1 to 100 rad/s at 25°C.

Human neuroblastoma SK-N-SH cells (kindly provided by prof. Adriana Bellucci, University of Brescia, Italy) were grown in complete medium composed of DMEM low glucose, supplemented with 10% fetal bovine serum, 1% penicillin/streptomycin, 1% L-glutamine, and 1% non-essential amino acids. Cells were maintained at 37°C under a humidified atmosphere of 5% CO_2_.

The cell viability was tested by MTT (3-(4,5-dimethylthiazol-2-yl)-2,5-diphenyl tetrazolium bromide) assay. Neuroblastoma cells were seeded into a 48-well plate (15000 cells/well) in complete medium. After 24 h, cells were treated with different concentrations (0.001–100 μM) of the compound of interest or with vehicle (1% DMSO). Treated and untreated cells were incubated for 24 h at 37°C under a humidified atmosphere of 5% CO_2_. Next, 30 μL of MTT (5 mg/ml) was added to the cells and incubated for 3 h at 37°C under a humidified atmosphere of 5% CO_2_. Afterward, formazan crystals were solubilized with acidified (0.1M HCl) isopropanol containing 10% Triton X-100. The optical density (OD) was determined at a wavelength of 570 nm using an Infinite 200Pro Tecan plate reader (Tecan Group Ltd.). Compounds were tested in triplicate. The cell viability was expressed as the percentage (%) of *viable cells* relative to the vehicle (100%). All experimental data were shown as mean ± SD. Data were analyzed by a one-way non-parametric ANOVA Kruskal–Wallis test with Dunnett’s correction for multiple comparison using Prism Software. The statistical significance was set at *p* < 0.05.

## 3 Results and Discussion

### 3.1 Synthesis of the FA/TPE Conjugates and Their Characterization

#### 3.1.1 Synthesis

**SCHEME 1:**
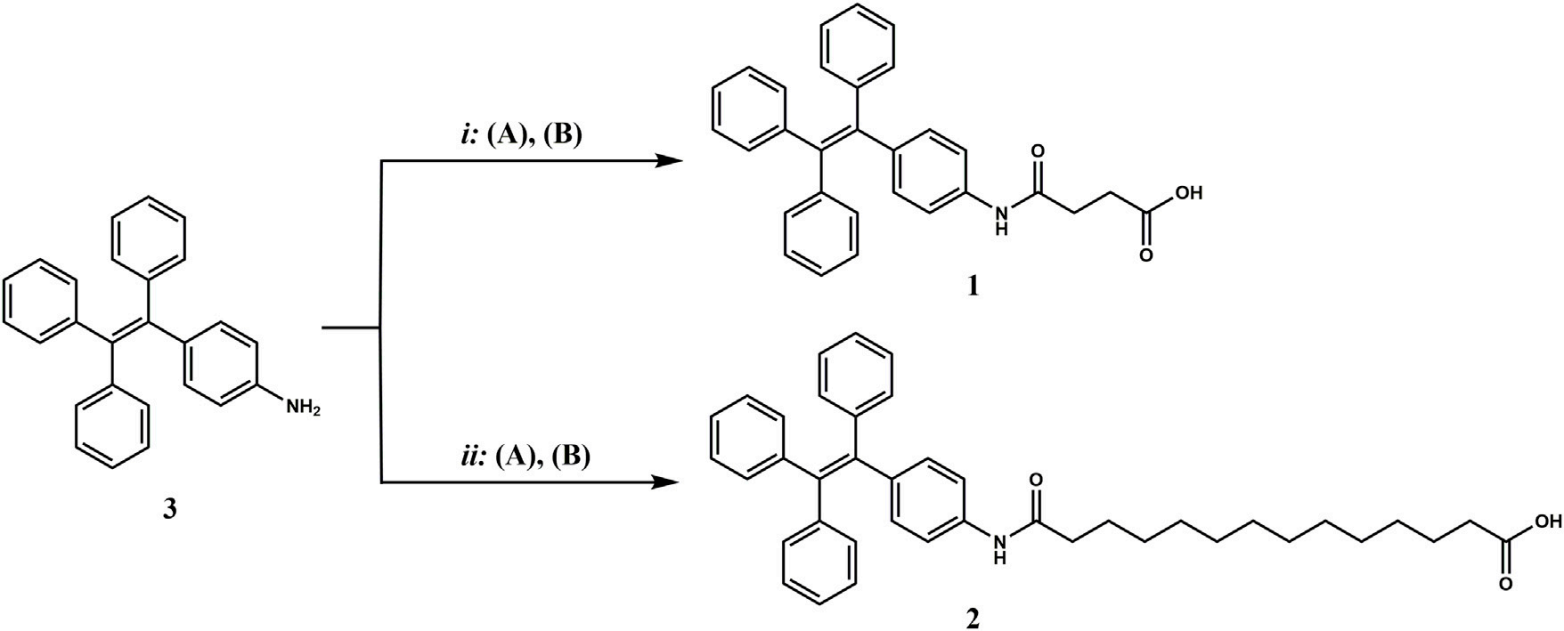
Synthesis of the FA/AIE conjugates, compounds **1** and **2**, starting from compound **3**, 4-amino-TPE. *i*: **(A)** succinic anhydride, compound **3**, DIEA in dioxane, reflux, 2 h; **(B)** room temperature (rt), overnight (o.n.).; *ii*: **(A)** tetradecanedioic acid, EDC hydrochloride in DCM, 0°C, 30 min; **(B)** compound **3**, DIEA in DCM, rt, o.n.

Compounds **1** and **2**, functionalized with succinic and tetradecanedioic acids, respectively, were synthesized starting from 4-amino-TPE (Scheme 1, compound **3**) obtained following a general protocol (reported in the SI). In the case of the shorter FA/TPE conjugate, the reaction was carried out in basic conditions, using DIEA as base in the presence of a slight excess of succinic anhydride in dioxane, to yield compound **1** in 65.27% (see the SI for procedure details; Supplementary Figure S1). Regarding the synthesis of the longer conjugate, compound **2**, the coupling reaction between compound **3** and tetradecanedioic acid was performed using EDC as a coupling reagent added in substoichiometric ratio, in order to activate only a carboxyl group. The desired product **2** was obtained in a 31.95% yield (see the SI for procedure details; Supplementary Figure S2).

#### 3.1.2 Fluorescence Spectroscopy

The photophysical properties of the synthesized AIEgens have been studied in dilute solutions, neat solid state, and as aggregate forms obtained from solvent/water mixtures, adding trifluoroacetic acid (TFA) mixture to avoid issues of carboxylic acid dissociation/amine-ammonium equilibrium.

**FIGURE 1:**
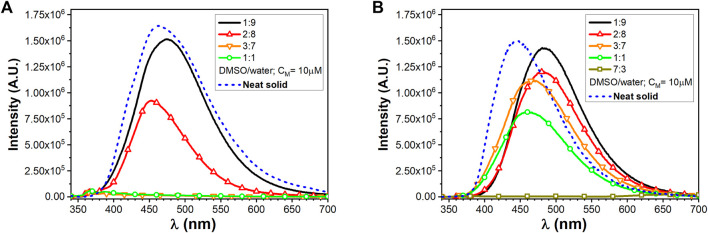
Emission spectra of TPE/FA conjugates, **(A)** for compound **1** and **(B)** for compound **2**, at different DMSO/water ratios (C_M_ = 10 µM) and in the presence of TFA together with solid-state neat emission.

In dilute solutions, the UV–vis absorptions of compounds **1** and **2** in dichloromethane (DCM) and dimethyl sulfoxide (DMSO) show the presence of a high energy band around 250 nm and a second one around 320 nm characterized by a molar absorptivity of ca. 1.5⨯10^4^ M^−1^cm^−1^, typical of a π–π*-type transition of the conjugated TPE scaffold (Bolzoni et al., 2013) (Supplementary Figures S7A, S8A in the SI). As observed in Supplementary Figures S7B, S8B in the SI, no appreciable optical variation can be observed with/without a small amount of TFA, implying that the protonation state of carboxylic acid function did not affect the TPE absorption features. As expected, no emission can be detected in the diluted solution arising from the TPE excitation. On the contrary, intense photoluminescence emissions can be detected upon molecular aggregations. The photophysical properties of the self-assembled **1** and **2** homostructures were evaluated by a modified solvent displacement technique used to self-assemble other aromatic peptides (Reches and Gazit, 2003; Yuran et al., 2012; Bonetti et al., 2015; Bucci et al., 2017; Bucci et al., 2020). To trigger the self-assembly, a stock solution of compounds **1** and **2** in DMSO (1 mM) was diluted with different DMSO/water mixtures (containing a small amount of TFA) to a final concentration of 10 µM and characterized after an incubation of 5–10 min. The aggregates of compound **1** at a 2:8 DMSO/water ratio have to be aged up to 40 min to get a stable luminescence. Figure 1 shows the emission properties of the aggregates together with the emission of the pure neat solid sample of the two compounds. The solid microcrystalline sample of **1** behaves like an AIEgen and shows a broad bluish emission with a maximum at 465 nm (FWHM 5500 cm^−1^) and a quantum yield of emission (QY) of 0.25. The spectrum from the 1:9 DMSO/water mixture clearly provides a similar bluish emission centered at 474 nm and displays a QY = 0.25. Increasing the amount of the DMSO content until reaching a 2:8 DMSO/water ratio, the emission undergoes a slight shift toward the blue (λ_max_ = 453 nm) and a slightly decreased QY (0.21). On the contrary, by just increasing the DMSO to 30% (3:7 DMSO/water mixture), no emission is detectable.

The microcrystalline solid sample of **2**, featuring a longer alkyl chain as a substituent of the TPE core, shows emission with a maximum at 445 nm (FWHM 4900 cm^−1^) and a QY of 0.1. It is worth noting that, in contrast to **1**, the emission from the 1:9 DMSO/water mixture clearly provides a similar bluish-green emission centered at 485 nm while displaying the highest QY of 0.52. The 2:8 DMSO/water mixture displays a similar emission although the QY drops to 0.44. The full photophysical parameters for compounds **1** and **2** are, respectively, reported in Supplementary Tables S3, S4 in the SI. Clearly, the presence of the longer alkyl chain in **2** results in a more pronounced tendency to form stable and efficient emissive aggregates even at lower water content.

#### 3.1.3 Dynamic Light Scattering Analyses

**FIGURE 2:**
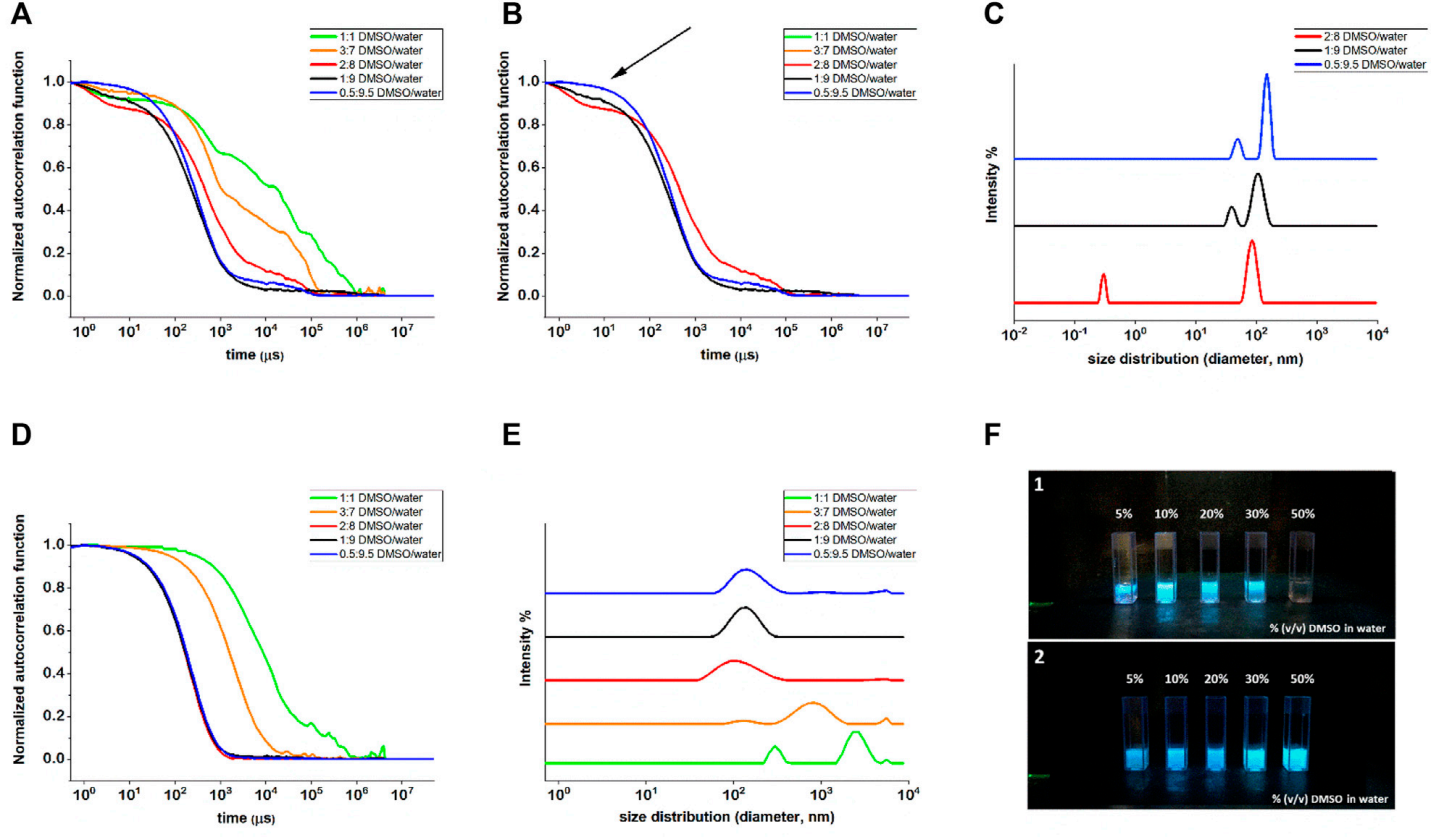
DLS measurements of compounds **1 (A**–**C)** and **2 (D,E)** in different DMSO/water mixtures. **(A)** Normalized autocorrelation functions of all the DMSO/water ratios, highlighting the noisy profiles of 1:1 and 3:7 DMSO/water ratios, whose fitting was not achievable; **(B)** normalized autocorrelation functions of compound **1** for 2:8, 1:9 and 0.5:9.5 DMSO/water ratios, where the arrow indicates the presence of 1:9 and 2:8 of small components that were not properly fitted by NNLS fitting; **(C)** intensity weighted size distribution for compound **1** in 2:8, 1:9, and 0.5:9.5 DMSO/water ratios. **(D)** Normalized autocorrelation functions for compound **2** and **(E)** intensity weighted size distribution for all the DMSO/water ratios for compound **2**. **(F)** Images of compounds **1** (F1 panel) and **2** (F2 panel) suspensions in water with increasing DMSO percentages. The pictures were taken under a UV light illumination (λ_ex_ = 365 nm).

Dynamic light scattering measurements were taken into account to get insights into the self-assembly tendency of compounds **1** and **2** as a function of the different DMSO/water ratios. Compound **1** showed a very poor scattering power when dissolved in 1:1 and 3:7 DMSO/water mixtures, and only when the water content was increased to reach a 2:8 DMSO/water ratio, the correlation function showed to be stable over several acquirements, suggesting that some aggregates were starting to form (Figure 2A).

**FIGURE 3:**
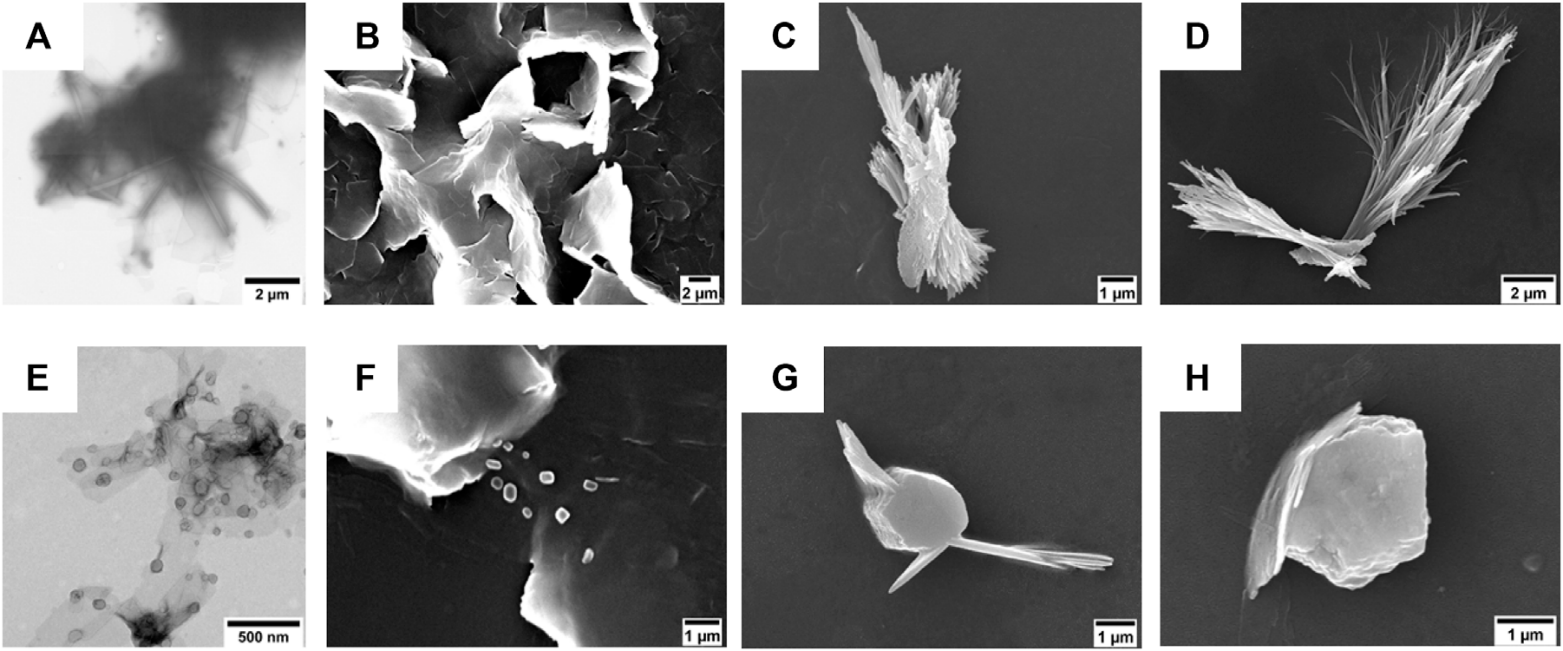
**(A,E)** TEM micrographs of **(A)** compound **1** (50 µM) and **(E)** compound **2** (50 µM). The scale bars are 1 μm **(A)** and 500 nm **(E)**. **(B–D)** SEM micrographs of compound **1** (50 µM) after its self-assembly in **(B)** 5% (v/v), **(C)** 10% (v/v), and **(D)** 20% (v/v) DMSO in water. Scale bars are 2 μm **(B,C)** and 1 μm **(D)**. **(F–H)** SEM micrographs of compound **2** (50 µM) after its self-assembly in **(F)** 5% (v/v), **(G)** 10% (v/v), and **(H)** 20% (v/v) DMSO in water. Scale bar is 1 μm.

Nevertheless, the fitting of the correlograms was poor, such that no reliable size distribution could be achieved. By further increasing the water content and passing from 1:9 to 0.5:9.5 DMSO/water ratio, the very first part of the correlogram changed, becoming less evident the starting flex (indicated with an arrow in Figure 2B) compatible with a very small component of the mixture. Good fittings of the curves were obtained only for the last two solvent ratios (1:9 and 0.5:9.5) that eventually led to the size distribution reported in Figure 2C) with two peaks, compatible with non-isotropic objects, as revealed by the TEM analysis for this molecule (see Figure 3A). Even compound **2** improved its scattering capacity when the non-solvent amount increased: while for 1:1 and 3:7 DMSO/water ratios, the correlograms were poor and the size distribution showed two populations possibly related to the undissolved solid material, starting from the DMSO/water ratio 2:8 on, the correlograms became stable and repeatable (Figure 2D). These findings suggested the formation of stable and regular self-assembled structures, and their fitting led to size distribution by intensity with one population only, centered at 140 ± 44 nm (Figure 2E, the upper traces). The obtained value of hydrodynamic diameter was in agreement with the TEM results (see Figure 3B). Moreover, the behavior of the two compounds seen by DLS nicely agrees with the fluorescence spectroscopy results, suggesting that for compound **2** the aggregates start to massively form in mixtures with a minor water content, as it can be macroscopically observed in the pictures of the different DMSO/water mixtures (Figure 2F).

#### 3.1.4 Electron Microscopy Analyses

The morphologies of the aggregates were investigated by TEM and SEM. In particular, the TEM analysis conducted on the suspensions of compounds **1** and **2** in 5% (v/v) DMSO in water showed the presence of tubular and lamellar structures for the shortest FA/TPE conjugate (Figure 3A) and the formation of spherical assemblies for the longest one (Figure 3E).

On the other hand, SEM investigations revealed that the obtainment of stable morphologies is strictly dependent on water concentration (Figures 3B–D,F–H).

In the case of compound **1**, at lower DMSO percentages, the formation of lamellar structure is evident (Figures 3B,C). This is probably due to the strong π−π interactions among the hydrophobic TPE moieties leading to a coalescence of the nanotubular assemblies into plates (Salimimarand et al., 2017). By increasing the DMSO amount (Figure 3D), it is indeed possible to observe the bundle of nanofibers characterized by an 85 nm inner diameter (Supplementary Figure S12D). The coalescence was also observed upon heating at 60°C (see Supplementary Figure S12 in the SI).

The elongation of the alkyl chain on compound **2** leads to a different *scenario* in which the presence of both globular and fibrillar structures is evident. In particular, the formation of bigger agglomerates was observed by increasing the amount of DMSO (Figures 3G,H). This change in the morphology could be ascribed to the longer hydrophobic chain that, being more flexible, could favor a more compact, globular shape.

The self-assembly behavior of both FA/TPE conjugates was ascertained by confocal microscopy (Supplementary Figure S16). The confocal images of the colloidal suspensions (10% (v/v) DMSO in water) confirmed the presence of spherical aggregates of fibers for compound **1** and bigger globular agglomerates for compound **2**.

### 3.2 FA/TPE Peptides: Synthesis and Characterization

#### 3.2.1 Synthesis

**SCHEME 2:**
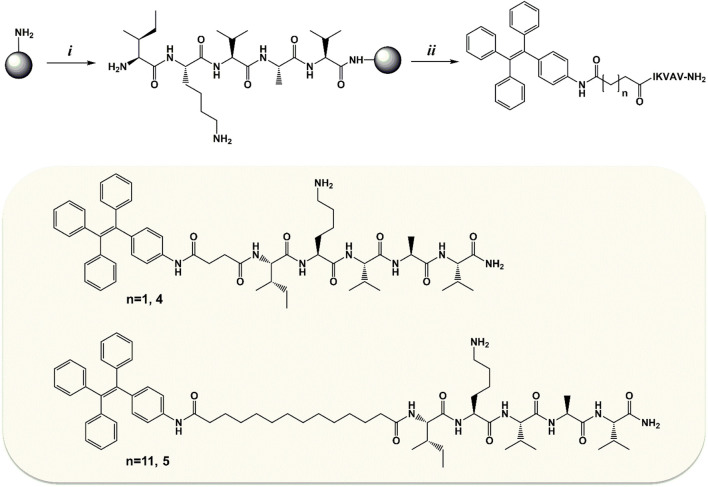
Synthesis of compounds **4** and **5**. *i*: the 5-mer sequence of laminin (IKVAV-NH_2_) was synthesized manually by SPPS using the Fmoc/t-Bu strategy and Rink Amide AM resin (100–200 mesh, loading 0.7 mmol/g) as solid support. *ii*: the on-resin coupling with the TPE/FA conjugate (**2** for compound **4** or **3** for compound **5**) was performed o.n. at rt in DMF using as coupling system 3:2.5:4 equivalents of TPE/FA conjugate/HATU/DIEA. Once the Kaiser test confirmed the successful conjugation, the peptidyl-bound resin underwent cleavage, giving compound **4** or **5**.

Compounds **1** and **2** were then used as N-*terminus* capping agents for ultra-short peptides. In particular, the IKVAV sequence from laminin was selected. This sequence is able to induce fibril formation in water environment, and it has been used for the functionalization of polymeric hydrogels (Perera et al., 2019; Yin et al., 2021). Here, we wanted to investigate the effect of FA/TPE hybrids on IKVAV self–assembly. Our aim was to obtain new AIE peptide-based nanomaterials, through the combination of the luminogen feature of TPE, the self-assembly propensity of FAs, and the amyloidogenic 5-mer laminin sequence. Furthermore, the gelation ability of the so-obtained hybrid compounds was also investigated. To this aim, compounds **4** and **5** were synthesized by solid-phase peptide synthesis (SPPS) using the Fmoc/t-Bu strategy (Scheme 2).

#### 3.2.2 Fluorescence Spectroscopy

**FIGURE 4:**
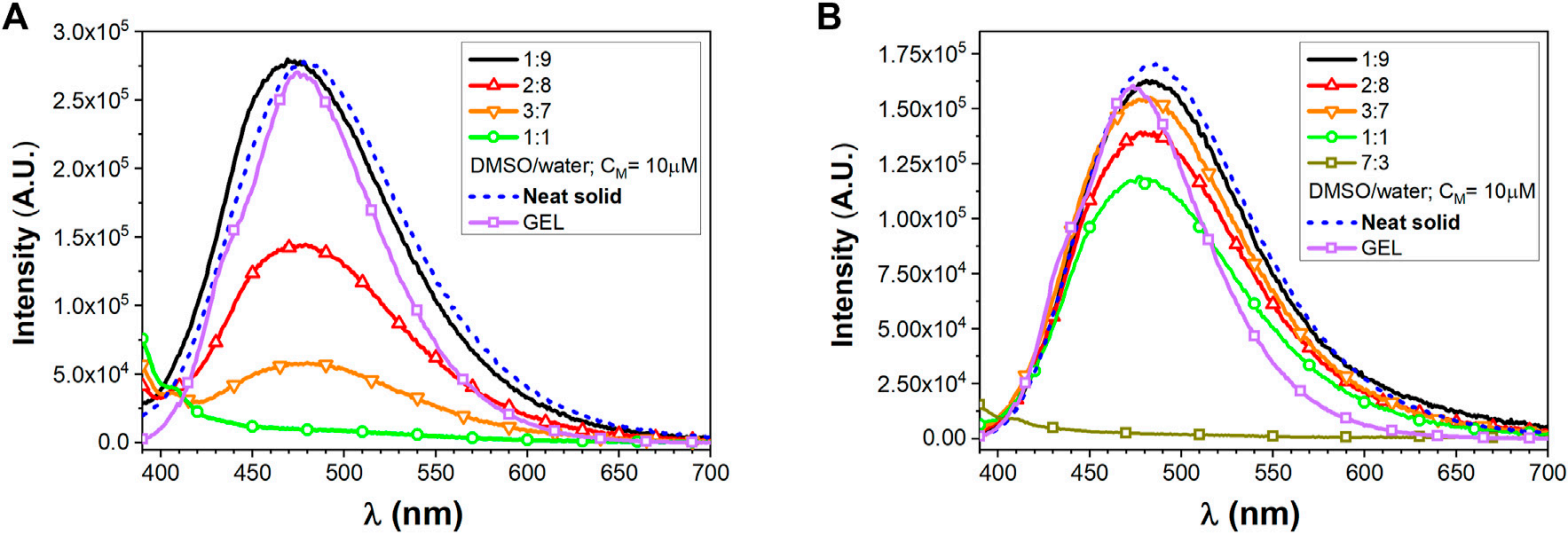
Emission spectra of peptide conjugates, **(A)** for compound 4 and **(B)** for compound 5, at different DMSO/water ratios (C_M_ = 10 µM) and in the presence of TFA together with solid-state neat emission and GEL emission.

As already discussed for precursors **1** and **2**, the new peptide conjugates have been investigated in solution and as aggregates, again in the presence of a trace amount of TFA. Absorption studies on compounds **4** and **5** were performed only in DMSO, since they are only partially soluble in DCM. As reported in Supplementary Figures S9A, S10A in the SI, the absorptions closely resemble those of the parent compounds **1** and **2** and they are correlated to the presence of the TPE chromophore. Derivatives **4** and **5** show a residual luminescence peaking at 365 nm in diluted DMSO solution, possibly ascribed to the TPE moiety (Figure 4). Intense photoluminescence emissions can be instead detected upon molecular aggregation. The photophysical properties of the self-assembled **4** and **5** homostructures were evaluated similarly to their parent systems **1** and **2**.

Derivatives **4** and **5** show a residual luminescence peaking at 365 nm in the diluted DMSO solution, possibly ascribed to the TPE moiety (Figure 4). Intense photoluminescence emissions can be instead detected upon molecular aggregation. The photophysical properties of the self-assembled **4** and **5** homostructures were evaluated similarly to their parent systems **1** and **2**.

The solid microcrystalline sample of **4** shows a broad bluish emission with a maximum at 479 nm (FWHM 4500 cm^−1^) and a QY of 0.38. The spectrum from the 1:9 DMSO/water mixture clearly provides a similar bluish emission centered at 470 nm, displaying a QY = 0.14. Increasing the amount of DMSO content as in a 2:8 DMSO/water mixture led to a reduction of the tendency to aggregate resulting in poorly emissive system with a QY of only 0.055.

The solid microcrystalline sample of **5**, featuring a longer alkyl chain as a substituent of the TPE core, shows emission with maximum at 484 nm (FWHM 4400 cm^−1^) and a QY of 0.42. The luminescence of the 1:9 and 2:8 DMSO/water mixtures provides a bluish-green aggregate emission centered at 482 nm displaying a high QY = 0.62–0.65.

#### 3.2.3 Secondary Structure Analysis

**FIGURE 5:**
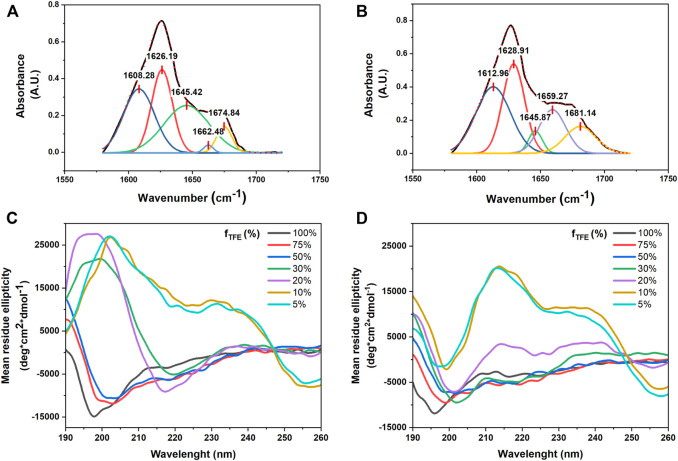
Selected region of ATR-FTIR spectra for compounds **4** and **5**: deconvoluted amide I peaks for compounds **4 (A)** and **5 (B)** in powder form. Both compounds are characterized by a mixture of conformations, with a predominant unordered structure for compound **4**, while compound **5** displays a higher propensity for the β-sheet conformation. CD signatures of **(C)** compounds **4** and **(D) 5**. All samples were prepared in various TFE–water mixtures at a concentration of 50 µM. The CD signal was converted to the mean residue ellipticity, which normalizes for peptide length and concentration.

The secondary structure of the synthesized FA/TPE peptides **4** and **5** was investigated by both ATR-IR and CD experiments (Figure 5). In the ATR-IR spectrum of compound **4** (Figure 5A), the deconvolution and the fitting of the amide I band showed a major peak in the amide I region at 1645.42 cm^−1^, indicating an unordered conformation. In the same region, the spectrum of compound **5** (Figure 5B) exhibited two major peaks at 1612.96 and 1628.91 cm^−1^, which correspond to a β-sheet conformation (Litvinov et al., 2012).

CD spectra of compounds **4** and **5** in different TFE/water mixtures (Figures 5C,D) showed that the secondary structure of the two peptides dramatically changed by increasing the percentage of water. The CD signatures of compounds **4** and **5** in 100% TFE showed a large negative band at from 190 to around 210 nm and small negative peaks at around 220 nm, which correspond to an unordered structure. By increasing the percentage of water until 50% (v/v), the CD signatures showed a positive peak at 190 nm, while the large negative band shifted to around 200 nm with small negative peaks at around 220 nm, suggesting a partial helical tendency for both the compounds. The CD signature of the two compounds in 20% (v/v) and 30% (v/v) TFE in water showed two opposite behaviors: while compound **4** showed an intense positive peak at 195 nm and a large intense negative band at around 220 nm, which correspond to a β-sheet conformation, compound **5** in 30% (v/v) was characterized by a positive peak at 190 nm and three negative peaks at 202, 219, and 225 nm, whereas in 20% (v/v), it revealed two slight Cotton effect bands approximately at 215 and 230 nm, which were due to the aromatic *π–π* effects of TPE. In addition, at the lowest concentration of TFE in water (20% (v/v), 10% (v/v), and 5% (v/v) for compound **4** and the latter two for compound **5**), the CD signatures showed a bisignated Cotton effect signal, approximately at 210 and 230 nm for compound **4** and approximately at 220 and 240 nm for compound **5**, indicating strong *π–π* stacking of TPE chromophores in water (Chu et al., 2018).

The tendency to self-assemble compounds **4** and **5** was also ascertained by the DLS investigation. The DLS measurements were carried out on the two compounds dissolved in 50 μM 0.5:0.95 DMSO/water mixture, after being aged for 48 h. The results showed a good scattering level, indicating the presence of some relevant nanostructures in suspension. The two species showed similar intensity-weighted size distribution profiles, presenting two peaks centered at 204 ± 27 nm and 996 ± 171 nm in the case of compound **4** and 359 ± 54 nm and 1230 ± 238 nm for compound **5** (Supplementary Figure S11). The double distribution is due to the non-isotropic shape of the self-assembled structures.

#### 3.2.4 Electron Microscopy Analyses

**FIGURE 6:**
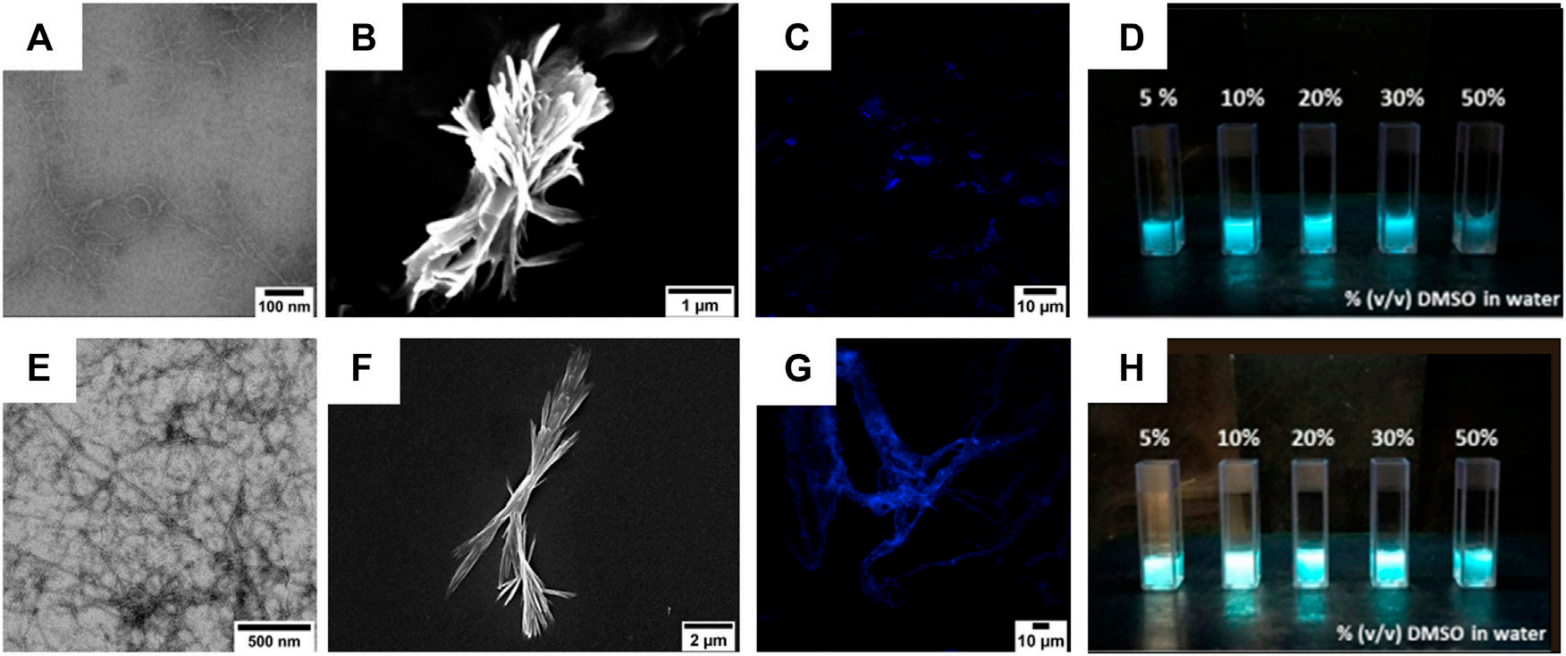
**(A,B)** TEM **(A)** and SEM **(B)** micrographs of compound **4** (50 µM) after its self-assembly in 1:9 (v/v) DMSO/water mixture. Scale bars are 100 nm **(A)** and 1 µm **(B)**. **(E,F)** TEM **(E)** and SEM **(F)** micrographs of compound **5** (50 µM) after its self-assembly in 1:9 (v/v) DMSO/water mixture. Scale bars are 200 nm **(E)** and 2 µm **(F)**. **(C,G)** Confocal images of compounds **4 (C)** and **5 (G)** after their self-assembly in a 1:9 (v/v) DMSO/water mixture. Scale bars are 10 µm. **(D,H)** Images of compounds **4 (D)** and **5 (H)** suspensions in water with increasing DMSO percentages. The pictures were taken under UV light illumination (λ_ex_ = 365 nm).

TEM and SEM analyses of nanocolloidal suspension of compound **4** in 10% (v/v) DMSO in water showed the formation of fiber-like aggregates (Figures 6A,B). A similar behavior was also observed for compound **5** (Figures 6C,D). In particular, both TEM and SEM images showed the formation of belts that could assemble into fibers, suggesting that the amyloid tendency of the peptide sequence prevails on the FA and TPE contributions to self-assembly.

The colloidal suspensions (10% (v/v) DMSO in water) were also visualized by confocal microscopy, showing that compound **4** preferred self-assembling into short and fractal fiber aggregates, whereas compound **5** adopted a large and symmetric fiber network.

#### 3.2.5 Gel Formation and Evaluation

**FIGURE 7:**
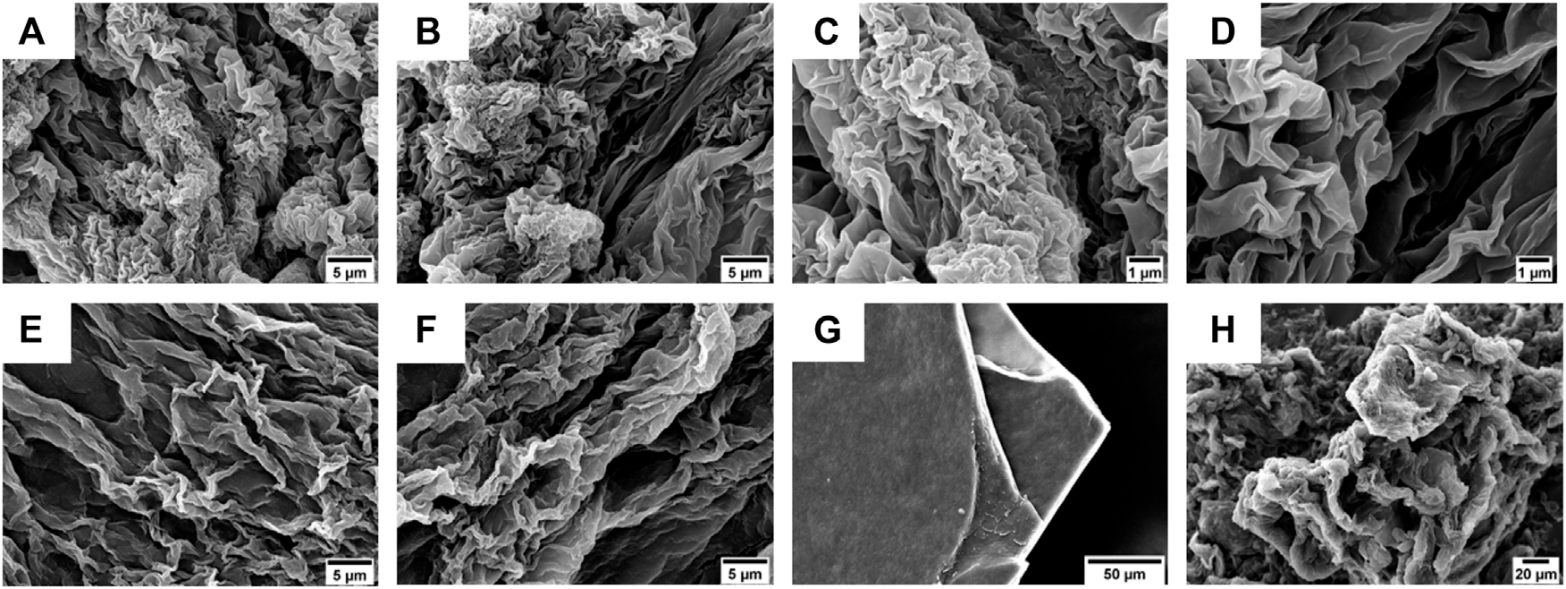
**(A–D)** SEM micrographs of the gel obtained from compound **4** (0.6% w/t) in 50% (v/v) CH_3_CN in 0.1 M NaHPO_4_ in water. Scale bars are 5 μm **(A,B)** and 1 μm **(C,D)**. **(E,F)** SEM micrographs of the gel obtained from compound **5** (0.6% w/t) in 50% (v/v) CH_3_CN in 0.1% TFA in water. Scale bar is 5 μm. **(G,H)** SEM micrographs of xerogel obtained from compounds **4 (G)** and **5 (H)** after slow evaporation at rt. Scale bars are, respectively, 50 µm **(G)** and 20 µm **(H)**.

Inspired from the previous results obtained by Jain *et al.* on laminin-derived lipophilic conjugates (Jain and Roy, 2019; Jain and Roy, 2020), compounds **4** and **5** were tested for gelation in various conditions, by varying: *i*) the pH of the organic/water mixtures, *ii*) the concentration of the gelators, and *iii*) the trigger for gelation (e.g., heating, sonication) (results not shown). The optimal conditions were 6 mM (0.6% w/t) in 50% (v/v) CH_3_CN in 0.1 M NaHPO_4_ in water (pH = 8.4) for compound **4**, while for compound **5** 5 mM (0.6% w/t) 50% (v/v) CH_3_CN in 0.1% TFA in water. The gelation of **4** was obtained at room temperature (rt) using alternate vortexing and sonication, allowing the solution undisturbed overnight (o.n.) to self-assemble in gel, while compound **5** required to be heated at 65°C for 30 min, after which the peptide self-assembled immediately in gel. The gel from compound **4** showed a flower-like fractal architectures when analyzed by SEM (Figures 7A–D).

On the other hand, the gel obtained using compound **5** as a gelator showed the presence of more extended lamellar structures (Figures 7E,F).

To better understand the morphology responsible for gel formation, we also analyzed the xerogel obtained from gels **4** and **5** after slowly evaporation at rt (Figures 7G,H). For compound **4**, lamellar multilayers were observed, while for **5,** a more disordered structure were present.

**FIGURE 8:**
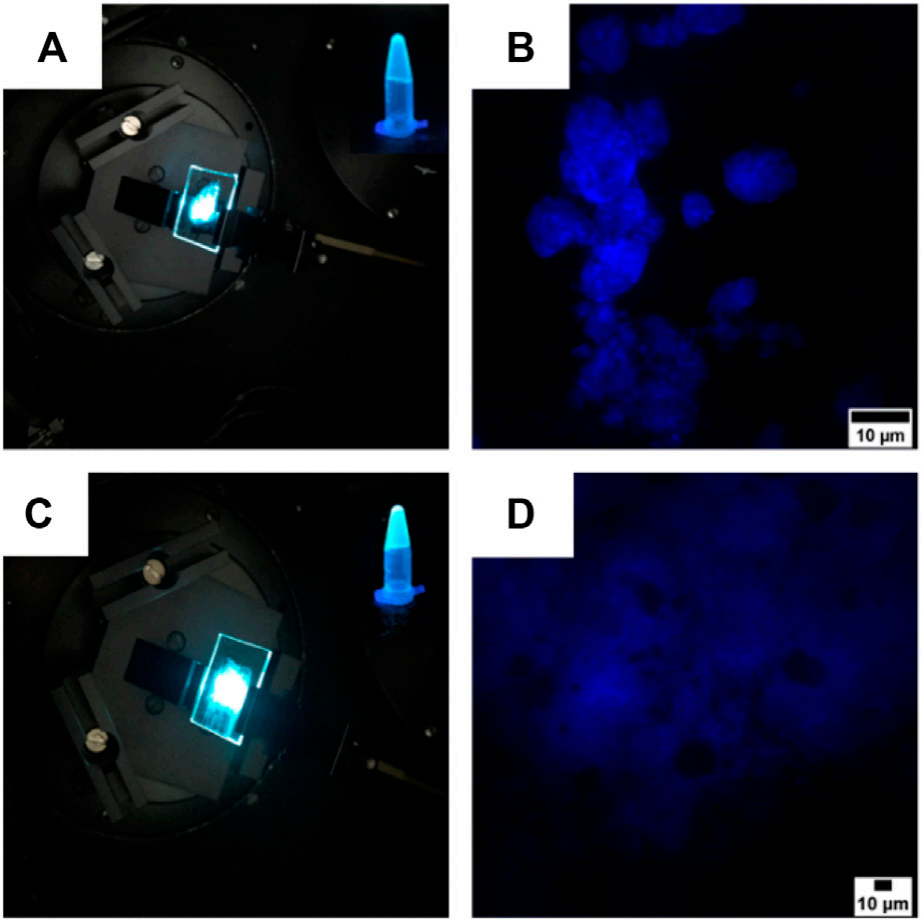
**(A,C)** Images of the gels obtained from compounds **4 (A)** and **5 (C)** spread on two coverslips with the superimposing photographs of the compounds in the gel state. The pictures of the cover slips were taken under a laser illumination (λ_ex_ = 325 nm), while the two superimposed photographs were taken under a UV light illumination (λ_ex_ = 365 nm). **(B,D)** Confocal images of the gels obtained from compounds **4 (B)** and **5 (D)**. Scale bars are 10 µm.

Gel formation also results in a luminescent material (Figures 8A,C), which was studied in analogy to the characterization already described for compounds **4** and **5**. The fluorescence traces are reported in Figure 6. Compound **4** displays an emission centered at 476 nm (FWHM 4050 cm^−1^) and a QY of 0.26. Compound **5** displays a bluish emission with a maximum at 475 nm (FWHM 3950 cm^−1^) and a QY of 0.38. For both compounds, the luminescence band and efficiency closely resemble those measured in the DMSO/water mixture as well as those obtained from the neat solids.

Confocal microscopy confirmed the architectures revealed by SEM, as the gel of compound **4** self-assembled into flower-like aggregates (Figure 8B), while the gel synthesized from compound **5** adopted a more flexible structure characterized by the presence of hollows (Figure 8D).

**FIGURE 9:**
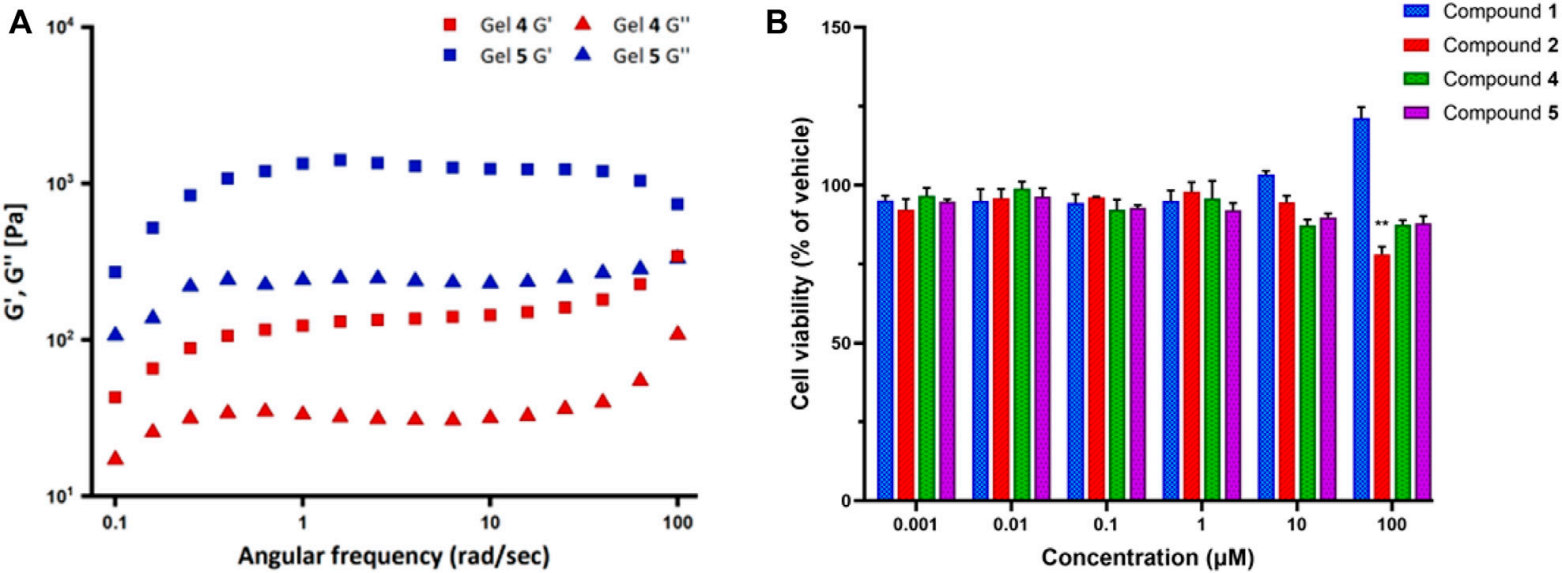
**(A)** Frequency sweep experiments for the gels synthesized from compounds **4** (Gel **4**) and **5** (Gel **5**). G′ is the storage modulus and G″ is the loss modulus. **(B)** The effect of compounds **1**, **2**, **4**, and **5** on cell viability by MTT assay. SK-N-SH neuroblastoma cells were treated with compound solutions ranging from 0.001 to 100 µM for 24 h. Data represent the mean ± SD of triplicate measurements. **p < 0.01 compound **2** versus vehicle.

Therefore, we tested the viscoelastic properties of the synthesized gels using oscillatory rheometry (Figure 9A). The frequency sweep experiments were performed from 0.1 to 100 rad/s with a strain of 0.8%.

The results showed that for both compounds **4** and **5**, the storage modulus (G′) is higher than the loss modulus (G″), thus indicating that both the gels have a predominant elastic feature over the viscous one. Moreover, both moduli have some dependence over frequency. Overall, compound **5** moduli are always far higher than those of compound **4**, G′ being approximately 1300 Pa versus 130 Pa in the 1–10 rad/s range. Such values, especially in the case of compound **5**, indicate that the gel is relatively stiff. At high frequencies, behaviors are different: G′ and G″ of compound **4** tend to increase, whereas compound **5** G′ decreases; therefore, at about 100 rad/s, the difference in the values of loss and elastic modulus between the two gels is lower than in the rest of the frequency range.

#### 3.2.6 Cytotoxicity

Finally, the cell viability of the FA/TPE hybrid compounds **1** and **2** and the conjugated peptides **4** and **5** were assessed by 3-(4,5-dimethylthiazol-2-yl)-2,5-diphenyltetrazolium bromide (MTT) assay. Neuroblastoma SK-N-SH cell line was used as the human neuronal model system. These cells are extensively used for both basic and applied research in biomedicine, including neurotoxicity (Cheung et al., 2009) and neurodegeneration (Xicoy et al., 2017). The cells were cultured for 24 h and then exposed to vehicle (1% DMSO) or the compound solutions in the concentration range from 0.001 to 100 µM for 1 day. The experimental results revealed that the viability of SK-N-SH cells significantly decreased upon treatment with 100 μM of compounds **2** (78.1%, respectively; *p* = 0.0047) when compared to vehicle (Figure 9B). Although the treatment of SK-N-SH cells with 10 and 100 μM of compound **1** demonstrated a slight increase in the cell viability, no significant changes were observed after treatment with compounds **1**, **4,** and **5** compared to vehicle.

## 4 Conclusion

In this work, hybrid molecules combining the AIE properties of TPE and the surfactant ability of fatty acids were synthesized and characterized. Starting from 4-amino-TPE, the functionalization with FAs of different lengths was performed through amide coupling. The emission properties and the self-assembly tendencies of the obtained compounds were studied by fluorescence, DLS, and electron microscopy (EM). Our results showed that the presence of a longer alkyl chain results in a more pronounced tendency to form stable and efficient emissive aggregates. The obtained compounds were then used as *N*-terminus capping agents in the development of peptide-based materials. In particular, the exploitation of the free carboxylic group led to the functionalization of the 5-mer laminin-derived IKVAV peptide. The conjugates self-assembled into luminescent fibrillary materials that were able to form supramolecular gels in the aqueous environment. Gels showed interesting rheological features, with a marked prevalence of elastic over loss modulus, thus indicating that they can be considered relatively stiff, especially in the case of compound **5**. Thus, the definition of compounds with the potential to supply a suitable substrate for *in vitro* models is relevant for a wide range of studies and applications.

## Data Availability

The original contributions presented in the study are included in the article/Supplementary Material; further inquiries can be directed to the corresponding author.
