# Supplementary material for: Fatty Acids/Tetraphenylethylene Conjugates: Hybrid AIEgens for the Preparation of Peptide-Based Supramolecular Gels
Source: Front Chem. 2022 Aug 8;10:927563. doi: 10.3389/fchem.2022.927563 (PMC9393247; doi:10.3389/fchem.2022.927563)
Supplement: Supplementary file 1 [file datasheet1.docx]

**Supporting information**

**Fatty acids/tetraphenylethylene conjugates: hybrid AIEgens for the preparation of peptide-based supramolecular gels**

Elisa Impresari^1^, Alberto Bossi^2^, Edoardo Mario Lumina^1^, Marco Aldo Ortenzi^3^, Josine Marie Kothuis^4^, Graziella Cappelletti^4^, Daniela Maggioni^5^, Michael S. Christodoulou^6^, Raffaella Bucci^1^, Sara Pellegrino^1*^

- 1. **Synthesis of compound 3 (*4-(1,2,2-triphenylvinyl)aniline*)**
  2. **Synthesis of compound 1 (*4-oxo-4-((4-(1,2,2-triphenylvinyl)phenyl)amino)butanoic acid*)**
  3. **Synthesis of compound 2 (*14-oxo-14-((4-(1,2,2-triphenylvinyl)phenyl)amino)tetradecanoic acid*)**
  4. **Solid-phase peptide synthesis (SPPS) of compounds 4 and 5**
  5. **NMR characterization of compounds 4 and 5**
  6. **Spectroscopic studies (FT-IR, Fluorescence)**
  7. **Dynamic Light Scattered (DLS) analyses of compounds 4, 5**
  8. **Self-assembly studies**
  9. **RP-HPLC, MS analyses of compounds 4 and 5**
  10. **NMR spectra**
  11. **References**

1. **Synthesis of compound 3 (*4-(1,2,2-triphenylvinyl)aniline*)**

Compound **3**, 4-amino-TPE, was synthesized according to McMurry reaction coupling (McMurry, 1989). Briefly, a mixture of zinc dust (3.2 g, 48,96 mmol, 9.67 eq) in THF (48 mL) was cooled to −10 °C by an ice-salt bath, then, TiCl_4_ (2.24 mL, 20.22 mmol, 4 eq) was added slowly. The mixture was stirred for 10 min and then refluxed for 2 h. To the mixture, cooled to −10 °C again, a THF solution (70 mL) of benzophenone (0.98 g, 5.36 mmol, 1.06 eq) and 4-aminobenzophenone (1 g, 5.06 mmol, 1 eq) was added slowly. The reaction was refluxed for 2 h under inert atmosphere. Once completed, the reaction was cooled to rt and then, quenched with a 10% (w/w) K_2_CO_3_ (100 mL) solution and kept under vigorous stirring overnight (on). The reaction was filtered, extracted with EtOAc and the collected organic layers were washed with brine and dried over anhydrous Na_2_SO_4_. After filtration and solvent evaporation in vacuum, the residue, an orange oil, was purified by flash silica column chromatography.

1. **Synthesis of compound 1 (*4-oxo-4-((4-(1,2,2-triphenylvinyl)phenyl)amino)butanoic acid*)**

Into a solution of succinic anhydride (0.0518 g, 0,518 mmol; 1.2 eq) and compound **3** (0,150 g; 0,432 mmol; 1 eq) in dioxane (12 mL), stirred for 10’ at 80°C, DIEA (0.151 mL, 0.864 mmol, 2eq) was added. The reaction was refluxed under vigorous stirring. Reaction completion was checked by TLC analysis (10:1 DCM/MeOH, R*_f_* 0.37). Once the reaction was completed, the raw product, obtained after evaporation, was washed with 5% (w/v) KHSO_4_ solution, brine and finally water. The target compound (0.126 g) was obtained as whitish solid (65.27% yield).

^1^H NMR (400 MHz, DMSO-*d_6_*) *δ* = 12.09 (1 H, s), 9.89 (1 H, s), 7.34 (2 H, d, *J* 8.7), 7.20 – 7.04 (9 H, m), 7.01 – 6.91 (6 H, m), 6.86 (2 H, d, *J* 8.7), 2.63 – 2.35 (4 H, m)

^13^C NMR (100 MHz, DMSO-*d_6_*) *δ* = 174.24, 170.46, 143.87, 143.80, 143.72, 140.71, 140.51, 138.16, 138.13, 131.53, 131.18, 131.13, 128.35, 128.25, 128.24, 126.98, 126.92, 126.88, 118.63, 31.48, 29.23

**
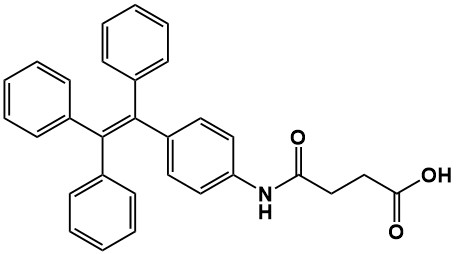
**ESI-MS (m/z) calculated for C_30_H_25_NO_3_ 448.19 m/z, found 446.73 [M-H]^-^.

**Figure S1**. Chemical structure of compound **1**.

1. **Synthesis of compound 2 (*14-oxo-14-((4-(1,2,2-triphenylvinyl)phenyl)amino)tetradecanoic acid*)**

In a 0.01 M solution of tetradecanedioic acid (0.179 g, 0.691 mmol, 1.2 eq) in DCM and few drops of DMF, EDC hydrocloride (0.066 g, 0.346 mmol, 0.6 eq) was added. The reaction was cooled at 0 °C and stirred for 1 h. Then, the solution was warmed to rt and the compound **3** (0.200 g, 0.575 mmol, 1 eq) was powdered. Into the new solution, DIEA (0.200 mL, 1.15 mmol, 2 eq) was added. Reaction completion was monitored by TLC analysis (20:1 DCM/MeOH, R*_f_* 0.52). Once the reaction was completed, the raw product, obtained after evaporation, was washed with 5% (w/v) KHSO_4_ solution, brine and finally, purified by flash silica column chromatography. The target compound (0.108 g) was obtained as yellowish solid (31.95% yield).

^1^H NMR (400 MHz, DMSO-*d_6_*) *δ* = 11.93 (1 H, s), 9.78 (1 H, s), 7.34 (2 H, d, *J* 8.3), 7.22 – 7.04 (10 H, m), 6.97 (5 H, dd, *J* 13.6, 7.0), 6.86 (2 H, d, *J* 8.2), 2.21 (4 H, dt, *J* 23.1, 7.4), 1.52 (4 H, dt, *J* 25.9, 6.9), 1.25 (16 H, d, *J* 7.4)

**
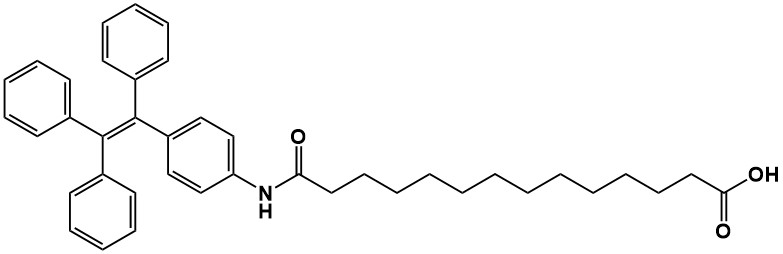
**^13^C NMR (100 MHz, DMSO-*d_6_*) *δ*= 174.96, 171.65, 143.89, 143.80, 143.74, 140.73, 140.49, 138.18, 138.16, 131.49, 131.17, 131.12, 128.33, 128.24, 128.22, 126.97, 126.91, 126.87, 118.75, 36.86, 34.15, 29.46, 29.43, 29.38, 29.25, 29.20, 29.12, 29.01, 25.56, 24.97

ESI-MS (m/z) calculated for C_40_H_45_NO_3_ 588.34, found 587.32 [M-H]^-^.

**Figure S2**. Chemical structure of compound **2**.

1. **Solid-phase peptide synthesis (SPPS) of compounds 4 and 5**

The peptides were synthesised on Rink Amide AM resin (100-200 mesh, 0.7 mmol/g), 0.1 mmol scale, using the manual solid-phase peptide synthesis (SPPS) Fmoc/t-Bu strategy.

0.143 g of the resin was swollen in DMF (1mL/100 mg of resin) for 40 min at rt and the mixture was gently stirred.

Each Fmoc group removal was performed by treatment of the resin with a solution of 20% piperidine (v/v) in DMF (5+15 min). Then, the resin was washed 3 times with DMF.

The coupling reactions were performed in DMF (1 mL/100 mg of resin) using as coupling system 5:5:6 equivalents of Fmoc-amino acid/HBTU/DIEA. The mixture was shaked for 25 min at rt and after that, the resin was washed 3 times with DMF.

The incorporation of each amino acid was confirmed by the Kaiser test (Kaiser *et al.*, 1970), indicating for all the couplings a complete reaction.

The N-*terminus* conjugation of the 5-mer precursor peptide to compound **1** and **2**, to give respectively compound **4** and **5**, was performed in DMF (0.5 mL/100 mg of resin) using as coupling system 3:2.5:4 equivalents of TPE/FA conjugate/HATU/DIEA. The coupling was carried out on, at rt and under gently shaking. The incorporation of the TPE/FA conjugate was monitored by Kaiser Test (Kaiser *et al.*, 1970), indicating for both the couplings a complete reaction.

Peptide cleavage from the resin and the concomitant deprotection of the side chain of Lys^2^ were carried out by treatment of the peptide-bound resin with a TFA/water/TIPS (95:2.5:2.5 v/v/v, 3mL) solution. The mixture was maintained at rt for 2h and 30 min, under gently shaking. The resin was washed with TFA (2 x 0.5 mL) and then, filtered. The crude product was precipitated by ice-cold Et_2_O (3 x 20 mL) and finally, suspended in DCM and evaporated *in vacuo*. The peptides **4** and **5** were analyzed by RP-HPLC and characterized by ESI-MS and NMR techniques.

1. **NMR characterization of compounds 4 and 5**

**Compound 4**

**Table S1.** NMR characterization of compound **4** (DMSO-d_6_, 293 K, 500 MHz).

| **AA** | **Atom** | **^1^H *δ*** | **Multiplicity**  ***J* (Hz)** | **ROESY** |
| --- | --- | --- | --- | --- |
| **N-*terminus***  **compound 1** | Ar CH | 6.92-7.01  7.06-7.22 | m  m |  |
|  | Csp^2^ |  |  |  |
|  | Csp^2^ |  |  |  |
|  | p-Ar CH | 6.87 | d (*J* =8.7 Hz) |  |
|  | p-Ar CH | 7.33 | d (*J* =8.7 Hz) | 9.87 NH^compound1^ |
|  | NH | 9.87 | s | 2.50 CH_2_^compound1^  7.33 CH^p-Ar^ |
|  | CO |  |  |  |
|  | CH_2_ | 2.50 | Overlapped with DMSO | 9.87 NH^compound1^ |
|  | CH_2_ | 2.46 | Overlapped with DMSO | 7.93 αNH^Leu1^ |
|  | CO |  |  |  |
| **Ile-1** | αNH | 7.93 | m | 2.46 CH_2_^compound1^ |
|  | αCH | 4.17 | m | 8.03 αNH^Lys2^  7.65 εNH_2_ ^Lys2^ |
|  | βCH | 1.72 | m |  |
|  | γCH_3_ | 0.81 | m |  |
|  | γCH_2_ | 1.09  1.43 | m  m |  |
|  | δCH_3_ | 0.81 | m |  |
|  | αCO |  |  |  |
| **Lys-2** | αNH | 8.03 | m | 4.17 αCH^Ile1^ |
|  | αCH | 4.29 | m | 7.63 αNH^Val3^ |
|  | βCH_2_ | 1.53  1.67 | m  m |  |
|  | γCH_2_ | 1.32 | m |  |
|  | δCH_2_ | 1.53 | m |  |
|  | εCH_2_ | 2.76 | m |  |
|  | εNH_2_ | 7.65 | m | αCH ^Ile1^ |
|  | αCO |  |  |  |
| **Val-3** | αNH | 7.63 | m | 4.29 αCH^Lys2^ |
|  | αCH | 4.18 | m | 8.06 αNH^Ala4^ |
|  | βCH | 1.97 | m |  |
|  | γCH_3_ | 0.83 | m |  |
|  | γCH_3_ | 0.83 | m |  |
|  | αCO |  |  |  |
| **Ala-4** | αNH | 8.06 | m | 4.18 αCH^Val3^ |
|  | αCH | 4.34 | m | 7.56 αNH^Val5^ |
|  | βCH_3_ | 1.20 | d (*J*=7.1 Hz) |  |
|  | αCO |  |  |  |
| **Val-5** | αNH | 7.56 | d (*J*= 9 Hz) | 4.34 αCH^Ala4^ |
|  | αCH | 4.10 | m | 7.02 NH^C-terminal^  7.33 NH^C-terminal^ |
|  | βCH | 1.96 | m |  |
|  | γCH_3_ | 0.84 | m |  |
|  | γCH_3_ | 0.84 | m |  |
|  | αCO |  |  |  |
|  | NH_2_ | 7.02  7.33 | m  d (*J* =8.7 Hz) | 4.10 αCH^Val5^ |

^13^C NMR (125 MHz, DMSO-*d_6_*) *δ* = 174.31, 173.16, 172.27, 171.97, 171.78, 171.66, 171.61, 170.92, 170.89, 158.46, 158.21, 143.88, 143.78, 143.72, 140.71, 140.54, 138.23, 138.08, 131.49, 131.16, 131.12, 128.33, 128.25, 128.23, 126.98, 126.90, 118.74, 57.78, 57.74, 57.49, 57.46, 52.87, 48.69, 39.21, 37.04, 36.98, 32.32, 31.49, 31.39, 31.15, 31.09, 31.06, 30.76, 30.40, 29.75, 29.47, 29.16, 27.01, 26.97, 24.76, 22.68, 19.69, 19.61, 18.36, 18.31, 18.29, 15.85, 11.54, 11.48.

**Compound 5**

**Table S2.** NMR characterization of compound **5** (DMSO-d_6_, 293 K, 500 MHz).

| **AA** | **Atom** | **^1^H *δ*** | **Multiplicity**  ***J* (Hz)** | **ROESY** |
| --- | --- | --- | --- | --- |
| **N-*terminus* Compound 2** | Ar CH | 6.93-7.05  7.07-7.19 | m  m |  |
|  | Csp^2^ |  |  |  |
|  | Csp^2^ |  |  |  |
|  | p-Ar CH | 6.86 | d (*J*=8.6 Hz) |  |
|  | p-Ar CH | 7.34 | m |  |
|  | NH | 9.78 | S | 7.34 CH^p-Ar^  2.23 CH_2_^Compound2^ |
|  | CO |  |  |  |
|  | CH_2_ | 2.23 | t (*J*=7.4 Hz) | 9.78 NH^Compound2^ |
|  | CH_2_ | 1.53 | m |  |
|  | CH_2_ | 1.28 | m |  |
|  | CH_2_ | 1.47 | m |  |
|  | CH_2_ | 2.06  2.16 | m | 7.85 αNH^Ile1^ |
|  | CO |  |  |  |
| **Ile-1** | αNH | 7.85 | d (*J*=8.6 Hz) | 2.16 CH_2_^Compound2^ |
|  | αCH | 4.14 | m | 8.04 αNH^Lys2^ |
|  | βCH | 1.73 | m |  |
|  | γCH_3_ | 0.83 | m |  |
|  | γCH_2_ | 1.11  1.42 | m  m |  |
|  | δCH_3_ | 0.83 | m |  |
|  | αCO |  |  |  |
| **Lys-2** | αNH | 8.04 | d (*J*=8.0 Hz) | 4.14 αCH^Ile1^ |
|  | αCH | 4.28 | m | 7.65 αNH^Val3^ |
|  | βCH_2_ | 1.53  1.64 | m  m |  |
|  | γCH_2_ | 1.32 | m |  |
|  | δCH_2_ | 1.52 | m |  |
|  | εCH_2_ | 2.74 | t (*J*=7.6 Hz) |  |
|  | εNH_2_ |  |  |  |
|  | αCO |  |  |  |
| **Val-3** | αNH | 7.65 | d (*J*=8.8 Hz) | 4.29 αCH^Lys2^ |
|  | αCH | 4.18 | m | 8.08 αNH^Ala3^ |
|  | C^β^H | 1.97 | m |  |
|  | γCH_3_ | 0.83 | m |  |
|  | γCH_3_ | 0.83 | m |  |
|  | αCO |  |  |  |
| **Ala-4** | αNH | 8.08 | d (*J*=7.4 Hz) | 4.19 αCH^Val3^ |
|  | αCH | 4.34 | m | 7.57 αNH^Val5^ |
|  | βCH_3_ | 1.20 | d (*J*=7 Hz) |  |
|  | αCO |  |  |  |
| **Val-5** | αNH | 7.57 | d (*J*=9 Hz) | 4.34 αCH^Ala4^ |
|  | αCH | 4.10 | m | 7.34 NH_2_^C-terminal^ |
|  | βCH | 1.96 | m |  |
|  | γCH_3_ | 0.86 | m |  |
|  | γCH_3_ | 0.86 | m |  |
|  | αCO |  |  |  |
|  | C-terminal NH_2_ | 7.02  7.34 | m  m | 4.10 αCH^Val5^ |

^13^C NMR (125 MHz, DMSO-*d_6_*) *δ* = 173.16, 172.79, 172.27, 171.78, 171.74, 171.65, 170.87, 143.89, 143.79, 143.74, 140.73, 140.51, 138.18, 131.49, 131.16, 131.12, 128.34, 128.24, 126.98, 126.90, 118.75, 57.76, 57.70, 57.29, 52.81, 48.65, 39.23, 36.85, 36.73, 35.56, 31.53, 31.19, 31.08, 29.50, 29.46, 29.41, 29.28, 29.22, 29.15, 29.05, 27.09, 25.86, 25.56, 24.83, 22.66, 19.69, 19.61, 18.38, 18.29, 15.85, 11.30.

1.

**Spectroscopic studies (FT-IR, Fluorescence)**

**Figure S3.** Full ATR-IR spectrum of compound **1**.





**Figure S4.** Full ATR-IR spectrum of compound **2**.







**Figure S5.** Full ATR-IR spectrum of compound **1**.

**Figure S6.** Full ATR-IR spectrum of compound **5**.





**Figure S7**. Absorption spectra of compound **1**: (A) molar absorptivities; (B) effect of addition of TFA; (C) excitation spectra.

**Table S3**. Summary of the photophysical parameters of compound **1**.

| **Compound 1** | **QY** | τ **(ns)** | τ_av_ **(ns)** |
| --- | --- | --- | --- |
| Neat Solid | 0.25 | 1.23(37%); 4.64(63%) | 4.17 |
| 1:9 DMSO/water | 0.25 | 0.36(8%); 1.79(45.9%); 5.08(46.1%) | 4.18 |
| 2:8 DMSO/water | 0.21 | 0.32(9%); 1.43(56%); 3.89(35%) | 2.94 |
| 3:7 DMSO/water | <0.01 | 0.11(13%); 1.50(41%); 5.19(46%) | 5.18 |





**Figure S8**. Absorption spectra of compound **2**: (A) molar absorptivities; (B) effect of addition of TFA; (C) excitation spectra.

**Table S4**. Summary of the photophysical parameters of compound **2**.

| **Compound 2** | **QY** | τ **(ns)** | τ_av_ **(ns)** |
| --- | --- | --- | --- |
| Neat Solid | 0.1 | 0.29(22%); 1.25(34%); 4.02(44%) | 3.40 |
| 1:9 DMSO/water | 0.52 | 0.21(6%); 1.55(34%); 4.37(60%) | 3.88 |
| 2:8 DMSO/water | 0.44 | 0.18(6%); 1.42(37%); 4.13(57%) | 3.63 |
| 3:7 DMSO/water | 0.44 | 0.15(7%); 1.49(39%); 4.35(54%) | 3.78 |
| 1:1 DMSO/water | 0.45 | 0.17(5%); 1.42(31%); 4.31(64%) | 3.91 |

**

Figure S9**. Absorption spectra of compound **4**: (A) molar absorptivities; (B) excitation spectra.

**Table S5**. Summary of the photophysical parameters of compound **4**.

| **Compound 4** | **QY** | τ **(ns)** | τ_av_ **(ns)** |
| --- | --- | --- | --- |
| Neat Solid | 0.38 | 0.47(6%); 1.98(41%); 4.95(53%) | 4.25 |
| DMSO Solution | 0.01 | 0.84(22%); 1.07(27%); 6.85(51%) | 6.40 |
| 1:9 DMSO/water | 0.14 | 0.31(7%); 1.85(41%); 5.61(52%) | 4.81 |
| 2:8 DMSO/water | 0.055 | 0.32(10%); 1.99(41%); 5.88 (49%) | 4.97 |
| 3:7 DMSO/water | 0.02 | 0.19(10%); 1.60(40%); 5.67(50%) | 4.91 |
| 1:1 DMSO/water | <0.01 | 0.19(5%); 1.61(39%); 4.90(56%) | 5.70 |
| GEL | 0.26 | 2.10(35.8%); 5.78 (64.2%) | 5.16 |





**Figure S10**. Absorption spectra of compound **5**: (A) molar absorptivities; (B) excitation spectra.

**Table S6**. Summary of the photophysical parameters of compound **5**.

| **Compound 5** | **QY** | τ **(ns)** | τ_av_ **(ns)** |
| --- | --- | --- | --- |
| Neat Solid | 0.42 | 0.48(5%); 2.22(38%); 5.32(57%) | 4.62 |
| 1:9 DMSO/water | 0.62 | 0.46(10%); 2.28(37%); 6.45(53%) | 5.37 |
| 2:8 DMSO/water | 0.65 | 0.21(6%); 1.89(36%); 6.05(58%) | 5.38 |
| 3:7 DMSO/water | (0.8) | 0.37(8%); 2.04(39%); 5.80(53%) | 4.98 |
| 1:1 DMSO/water | 0.50 | 0.19(5%); 1.61(39%); 4.90(56%) | 4.28 |
| GEL | 0.38 | 1.89(35.5%); 4.43(64.5%) | 3.95 |

1. **Dynamic Light Scattered (DLS) analyses of compounds 4 and 5**

The self-assembly of compound **4** and **5** was ascertained also by DLS investigation. The two compounds were dissolved in DMSO at the concentration of 1mM and then diluted with a 0.5:0.95 DMSO/water mixture to a final concentration of 50 µM. The mixtures were aged for 48 h and they showed a good scattering and similar size distribution by intensity with two peaks centred at 204 ± 27 nm and 997 ± 171 nm in the case of compound **4**, and 359 ± 54 nm and 1230 ± 238 nm for compound **5**. The double distribution is due to the non-isotropic shape of the self-assembled structures.





**Figure S11**. DLS measurements in 0.5:9.5 DMSO/water mixture of (A) compound **4** and (B) compound **5**, both analyzed at 50 μM.

1. **Self-assembly studies**

Compounds **1, 2, 4, 5** were dissolved in DMSO at the concentration of 1 mM. Then, the solutions were diluted with different DMSO/water mixtures to a final concentration of 50 µM. After overnight incubation the assemblies were studied by TEM and SEM analysis.

**
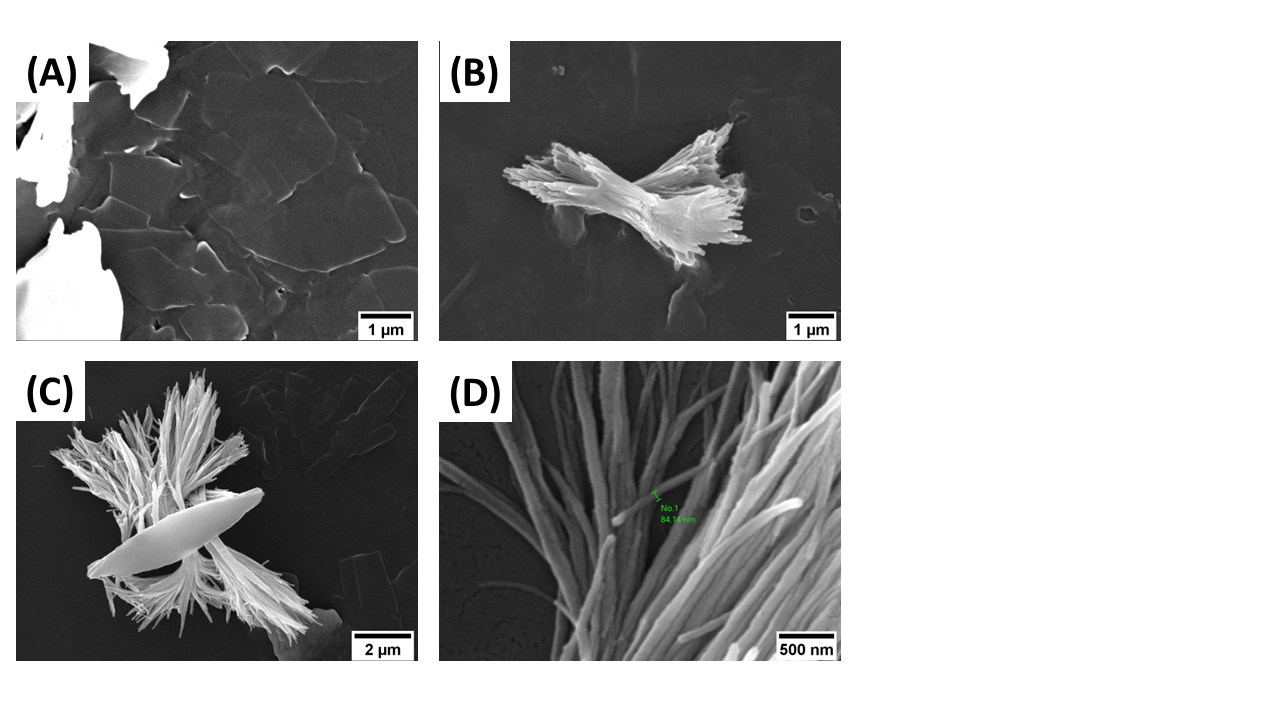
**


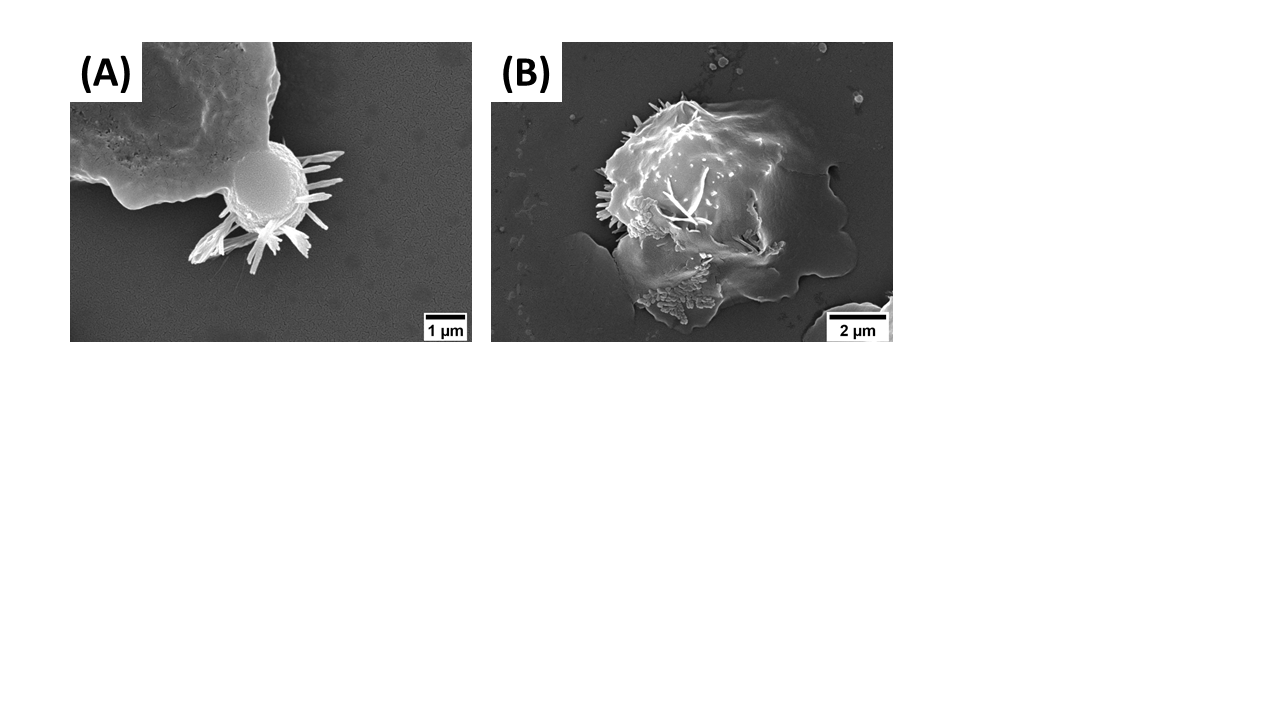


**Figure S12**. SEM micrographs of compound **1** (50 µM) after its self-assembly in: (A) 5% (v/v), (B) 10% (v/v) and (C, D) 20% (v/v) DMSO in water. Scale bars are 1 μm for (A, B), 2 μm for (C) and 500 nm for (D).

**Figure S13**. SEM micrographs of compound **1** (50 µM) after heating at 60°C. The self-assembly was performed in: (A) 10% (v/v) and (B) 20% (v/v) DMSO in water. Scale bars are 1 μm for (A) and 2 μm for (B).

**
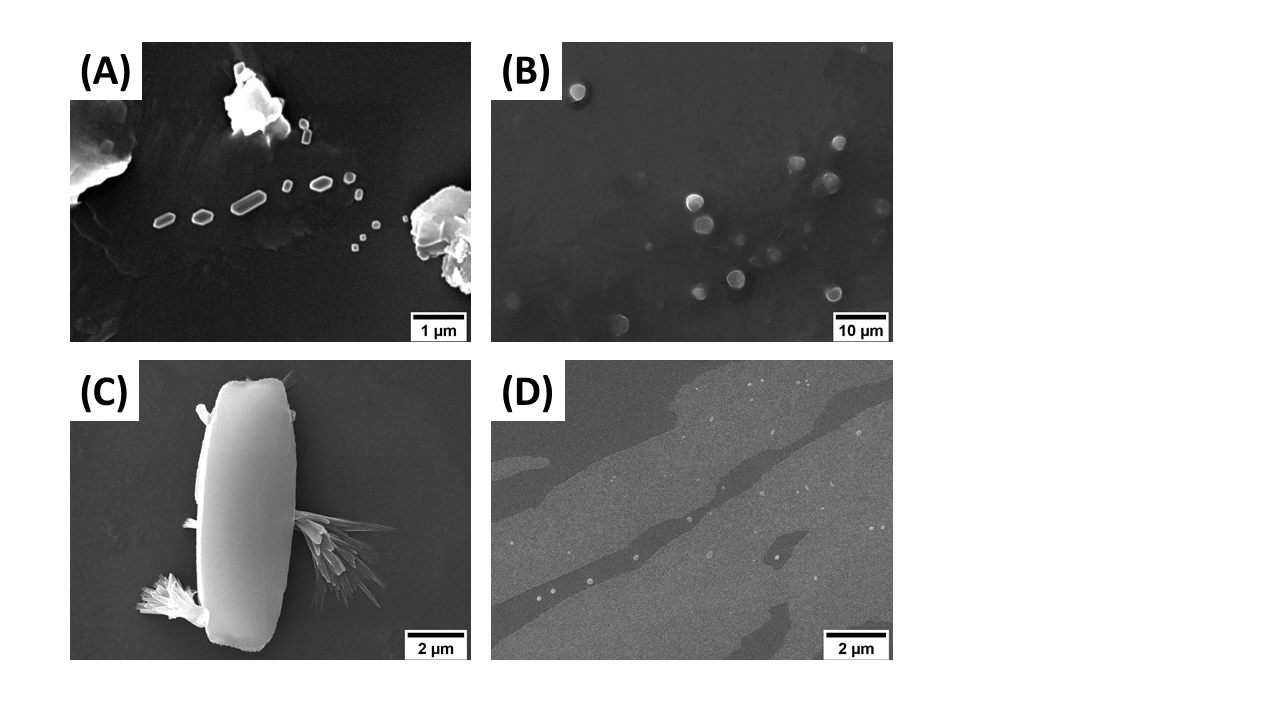
**

**Figure S14.** SEM micrographs of compound **2** (50 µM) after its self-assembly in: (A) 5% (v/v), (B) 10% (v/v), (C) 20% (v/v) and (D) 50% (v/v) DMSO in water. Scale bars are 1 μm for (A), 10 μm for (B) and 2 μm for (C, D).

**Figure S15**. SEM micrographs of compound **2** (50 µM) after heating at 60°C. The self-assembly was performed in: (A) 10% (v/v), (B) 20% (v/v) and (C) 50% (v/v) DMSO in water. Scale bars are 1 μm for (A), 5 μm for (B) and 10 μm for (C).


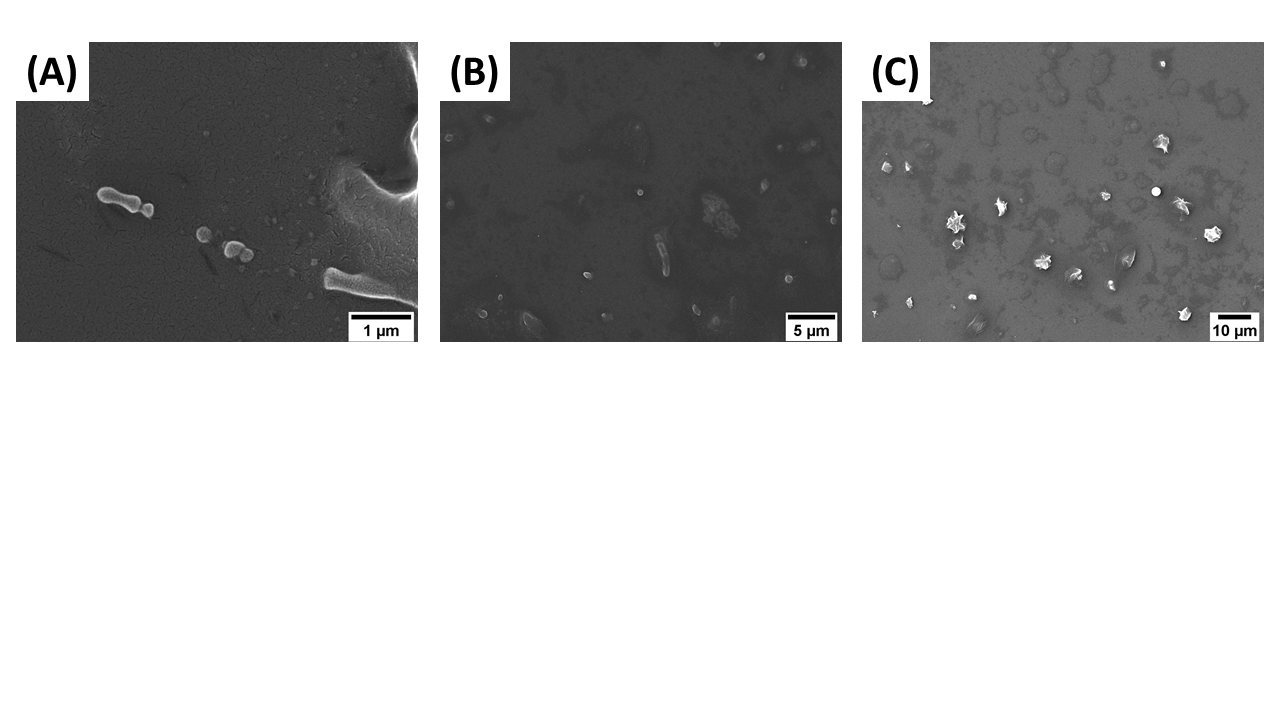


**
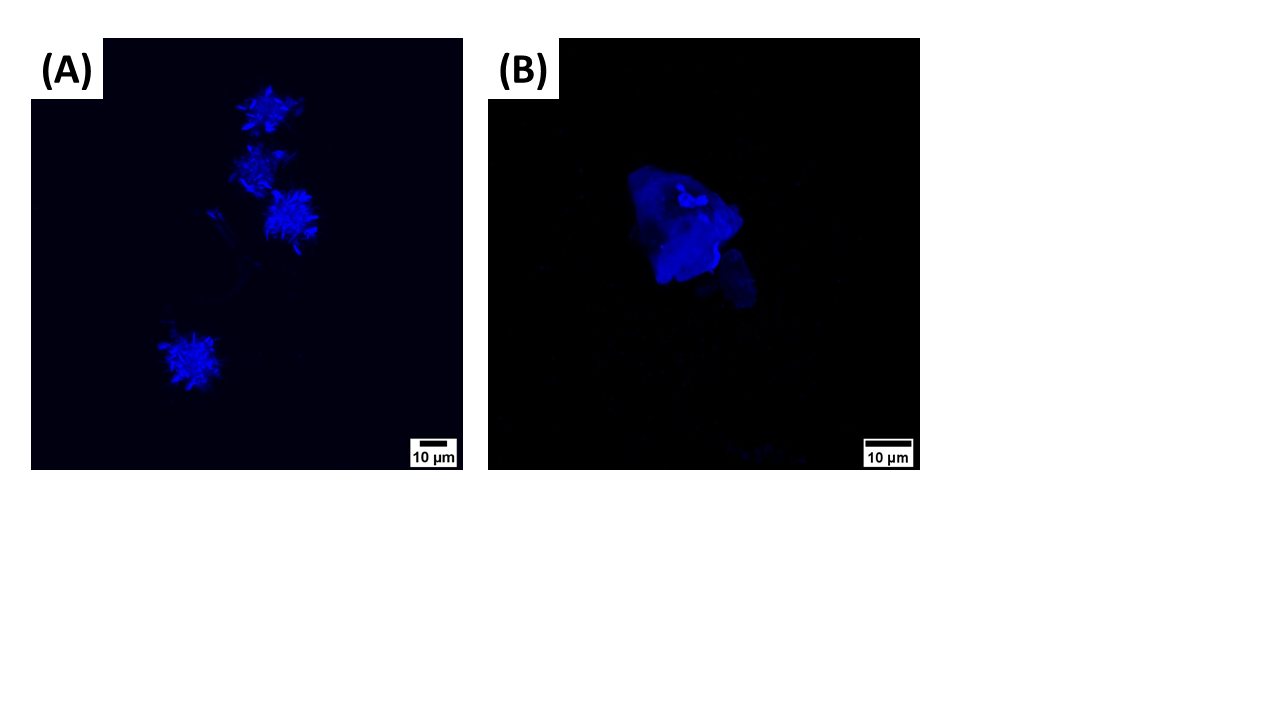
**

**Figure S16**. Confocal images of compound **1** (A) and compound **2** (B) after their self-assembly in 1:9 (v/v) DMSO/water mixture. Scale bars are 10 µm.

1. **

RP-HPLC and MS analyses of compounds 4 and 5**

**Figure S17**. UV chromatogram of compound **4**. Gradient: 40-100% of B in A in 20 min. 0.8 mL/min. A: 0.1% TFA in 100% H_2_O: B: 0.1% TFA in CH_3_CN.





**Figure S18.** ESI-MS spectrum of compound **4**. The peaks signed correspond to: [M+H]^+^ (957.66 m/z) and [M+Na]^+^ (979.75 m/z).





**Figure S19.** UV chromatogram of compound **5**. Gradient: 50-100% of B in A in 20 min. 0.8 mL/min. A: 0.1% TFA in 100% H_2_O: B: 0.1% TFA in CH_3_CN.





**Figure S20.** ESI-MS spectrum of compound **5**. The peaks signed correspond to: [M+H]^+^ (1098.64 m/z) and [M+2H]^2+^ (549.84 m/z).

1. **NMR spectra**

**Compound 1**

**
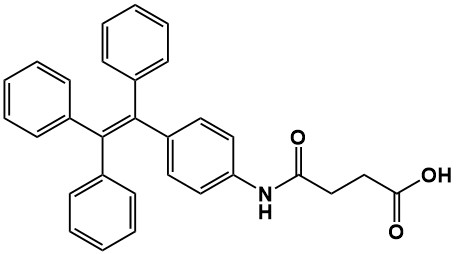
**

**Figure S21**. Chemical structure of compound **1**.

**
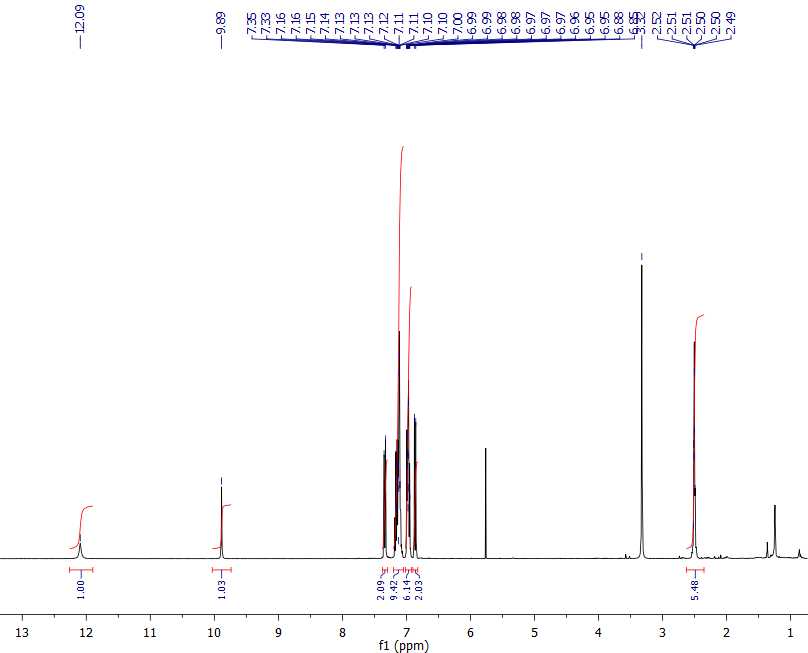
**

**Figure S22**. ^1^H NMR spectrum of compound **1** (400 MHz, DMSO-d_6_).

**
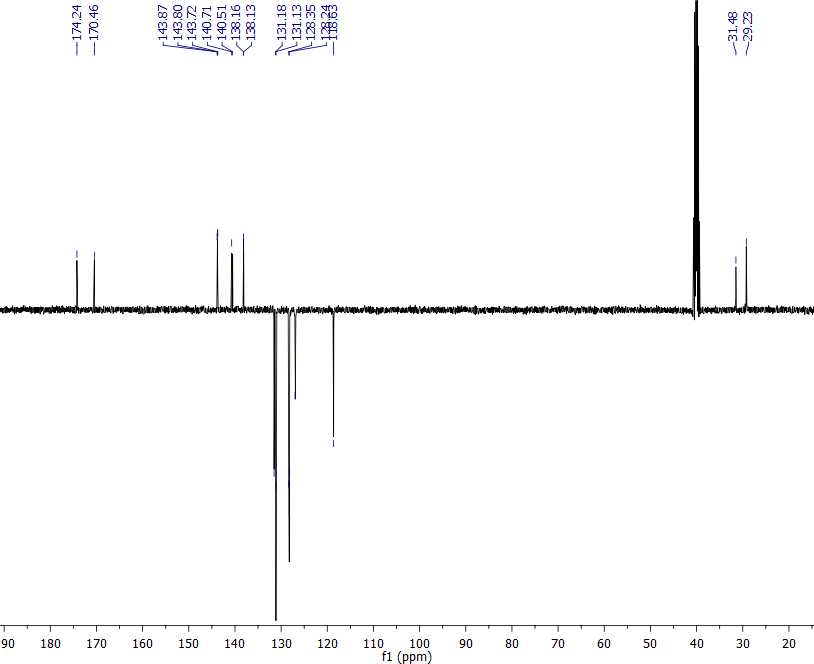
**

**Figure S23**. APT ^13^C-NMR spectrum of compound **2** (100 MHz, DMSO-d_6_).

**Compound 2**

**
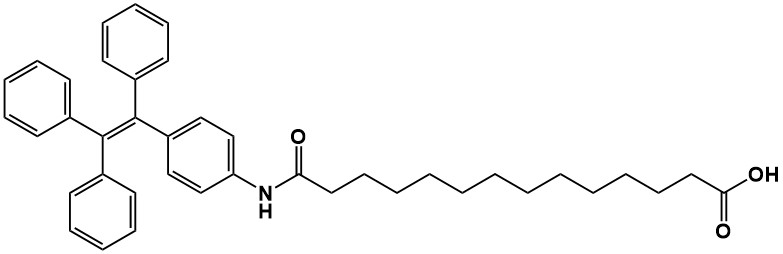
**

**Figure S24**. Chemical structure of compound **2**.

**
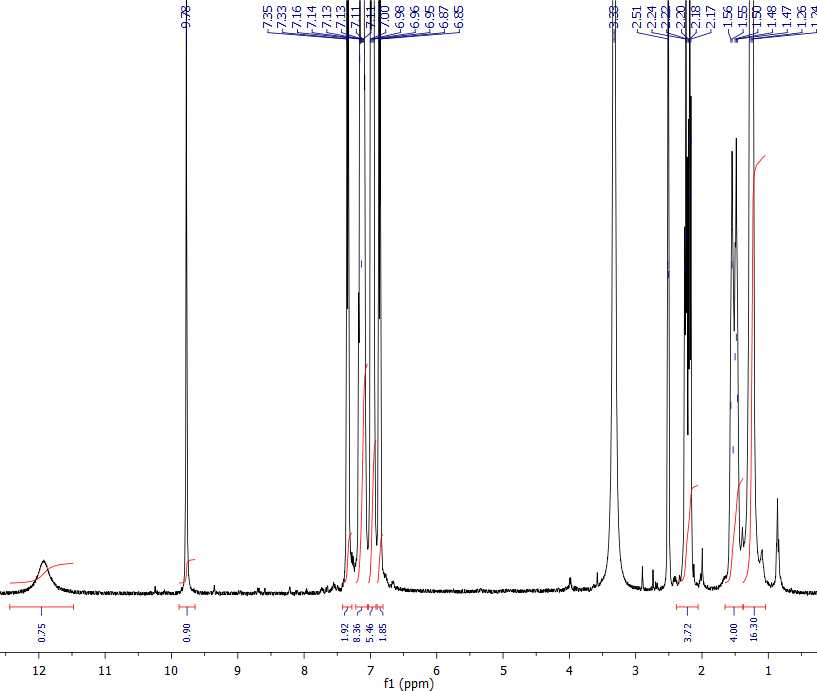
**

**Figure S25**. ^1^H NMR spectrum of compound **2** (400 MHz, DMSO-*d_6_*).

**
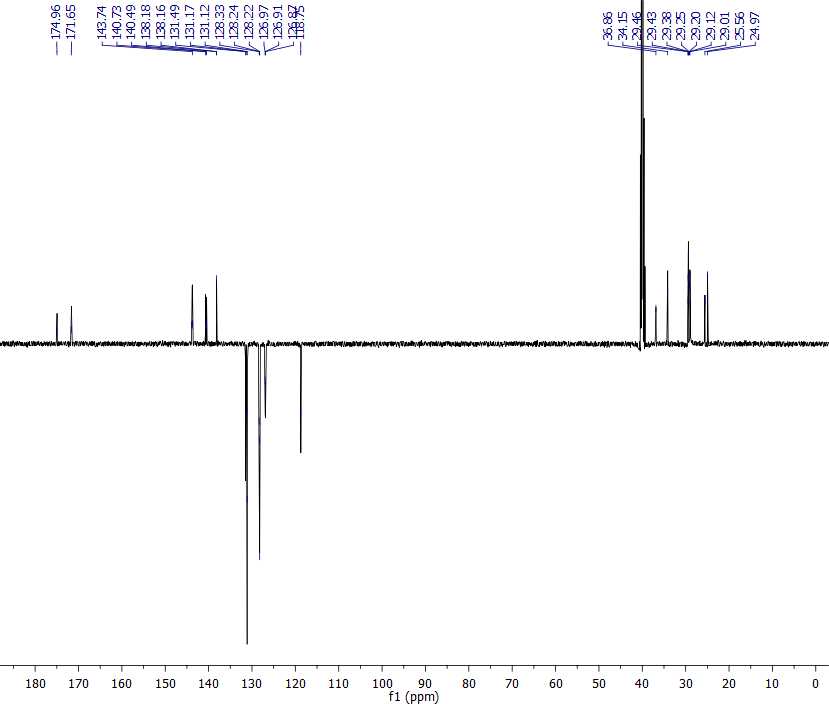
**

**Figure S26**. APT ^13^C NMR spectrum of compound **2** (101 MHz, DMSO-d_6_).

**Compound 4**

**
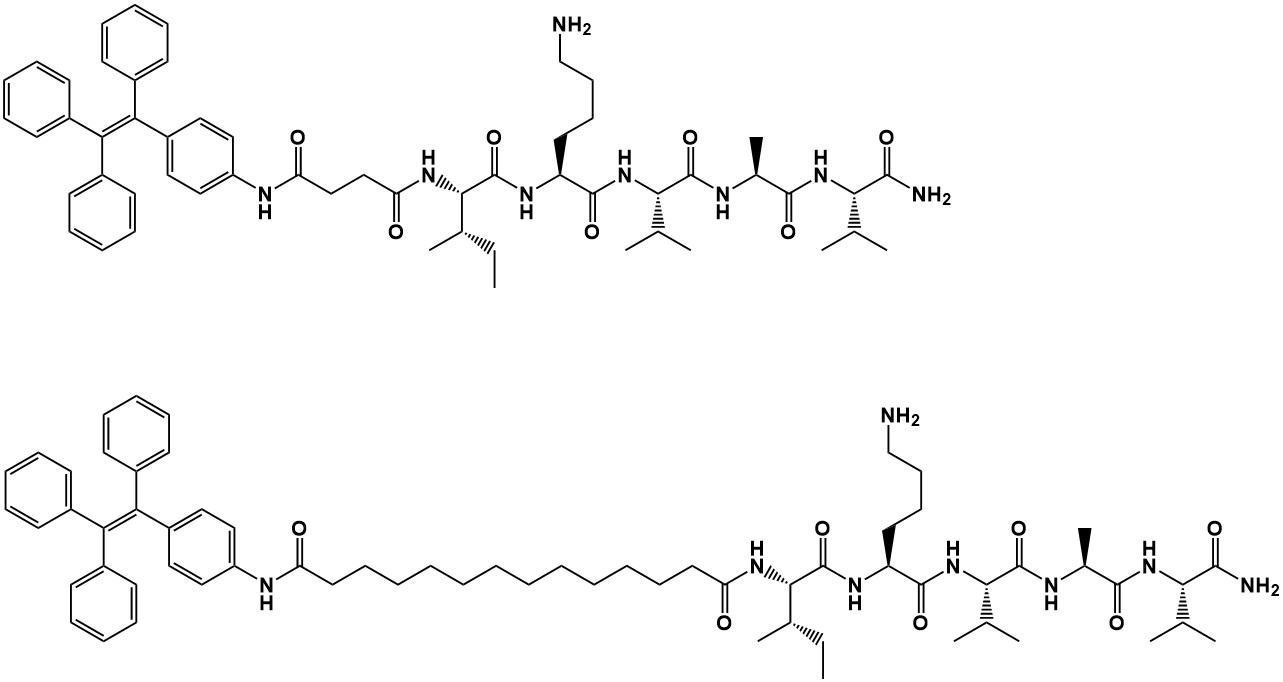
**

**Figure S27**. Chemical structure of compound **4**.

**
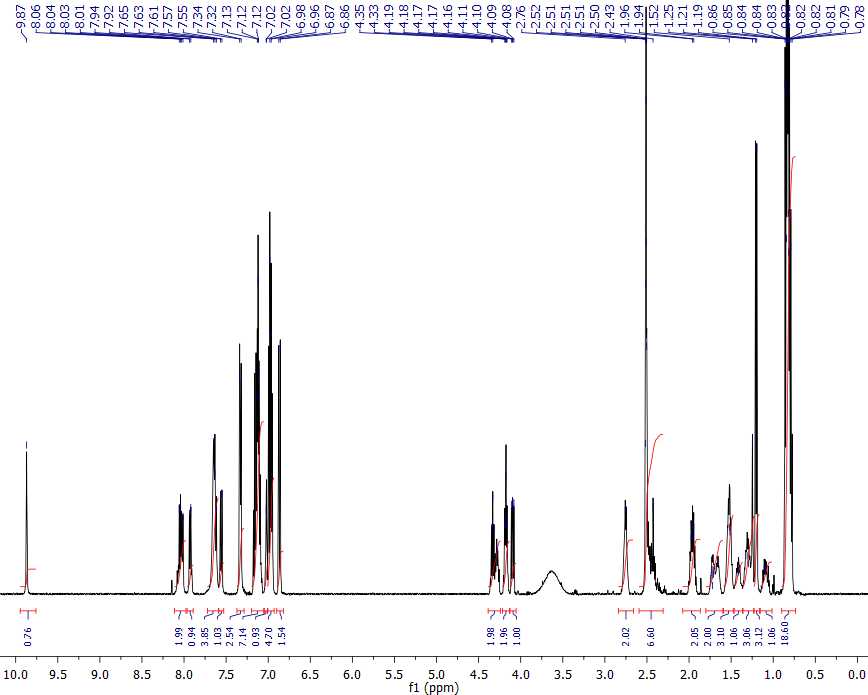
**

**Figure S28**. ^1^H NMR spectrum of compound **2** (500 MHz, DMSO-d_6_).

**
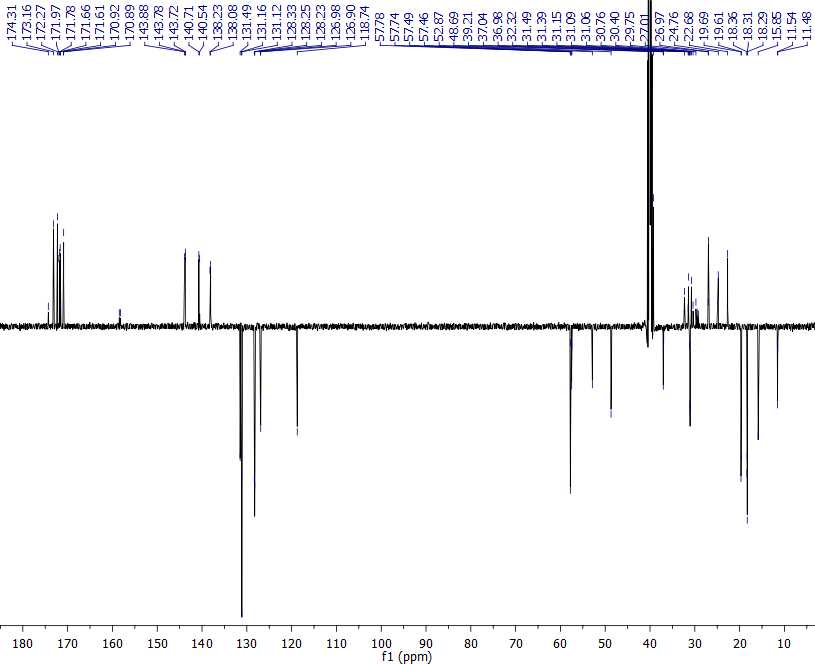
**

**Figure S29**. APT ^13^C NMR spectrum of compound **4** (125 MHz, DMSO-d_6_).

**
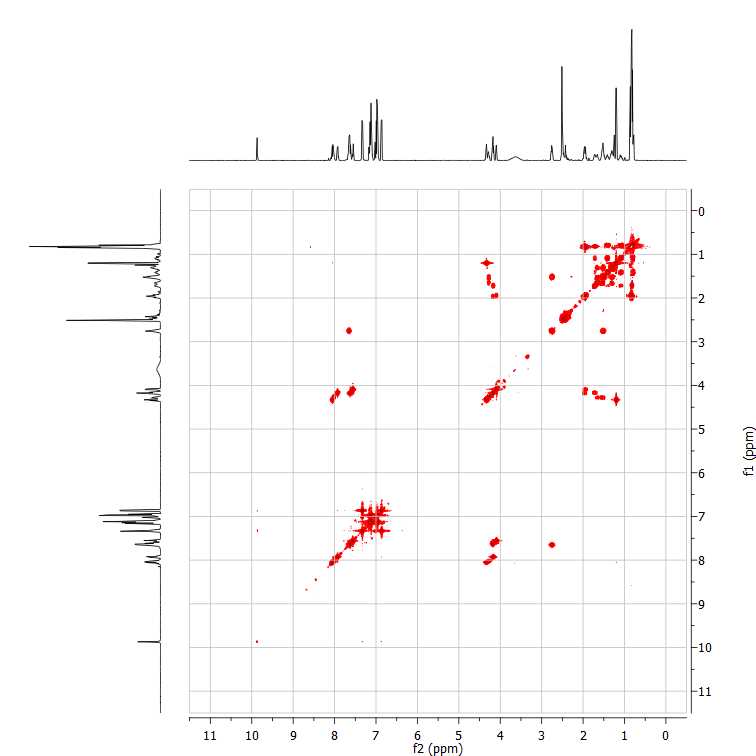

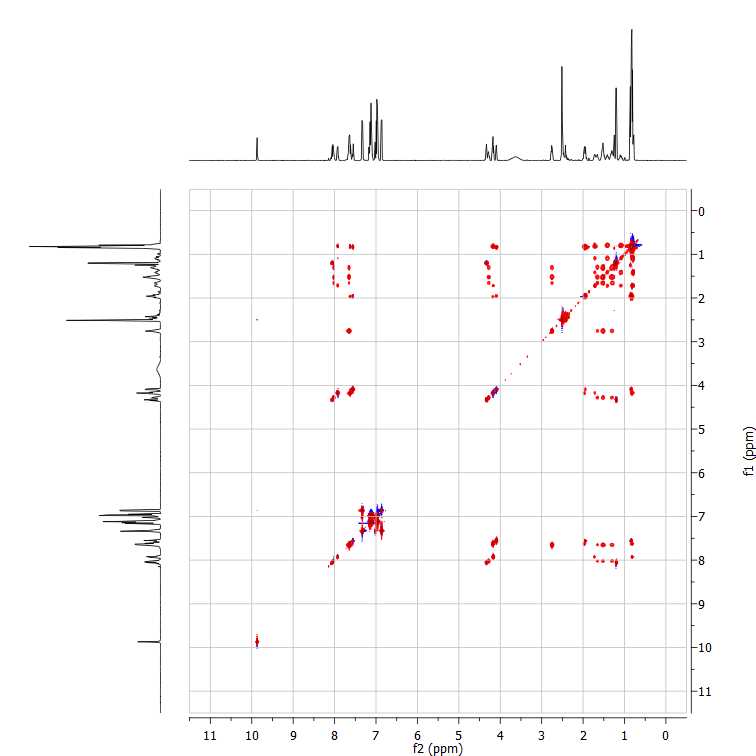
**

**Figure S30.** ^1^H,^1^H-COSY spectrum for compound **4** (500 MHz, DMSO-d_6_).

**Figure S31.** ^1^H,^1^H**-**TOCSY spectrum for compound **4** (500 MHz, DMSO-d_6_).

**
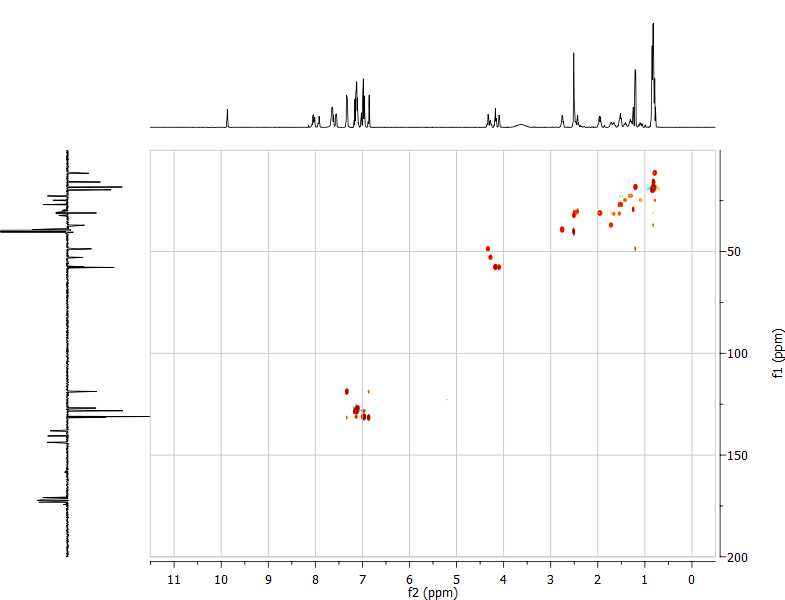

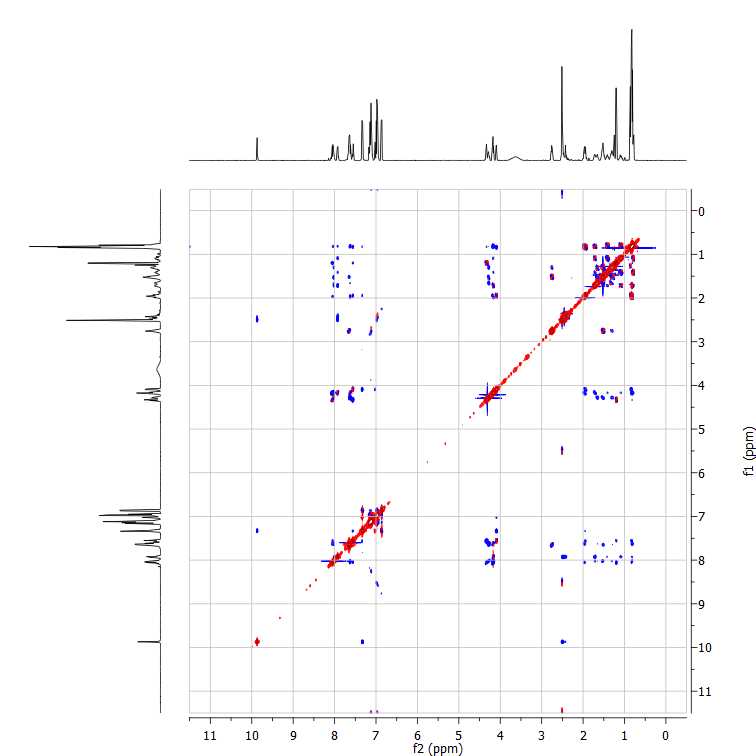
**

**Figure S32.** ^1^H,^1^H**-**ROESY spectrum for compound **4** (500 MHz, DMSO-d_6_, 200 ms).

**Figure S33.** ^1^H,^13^C**-**HSQC spectrum for compound **4** (500 MHz, 125 MHz, DMSO-d_6_).

**Compound 5**

**Figure S34**. Chemical structure of compound **5**.

**
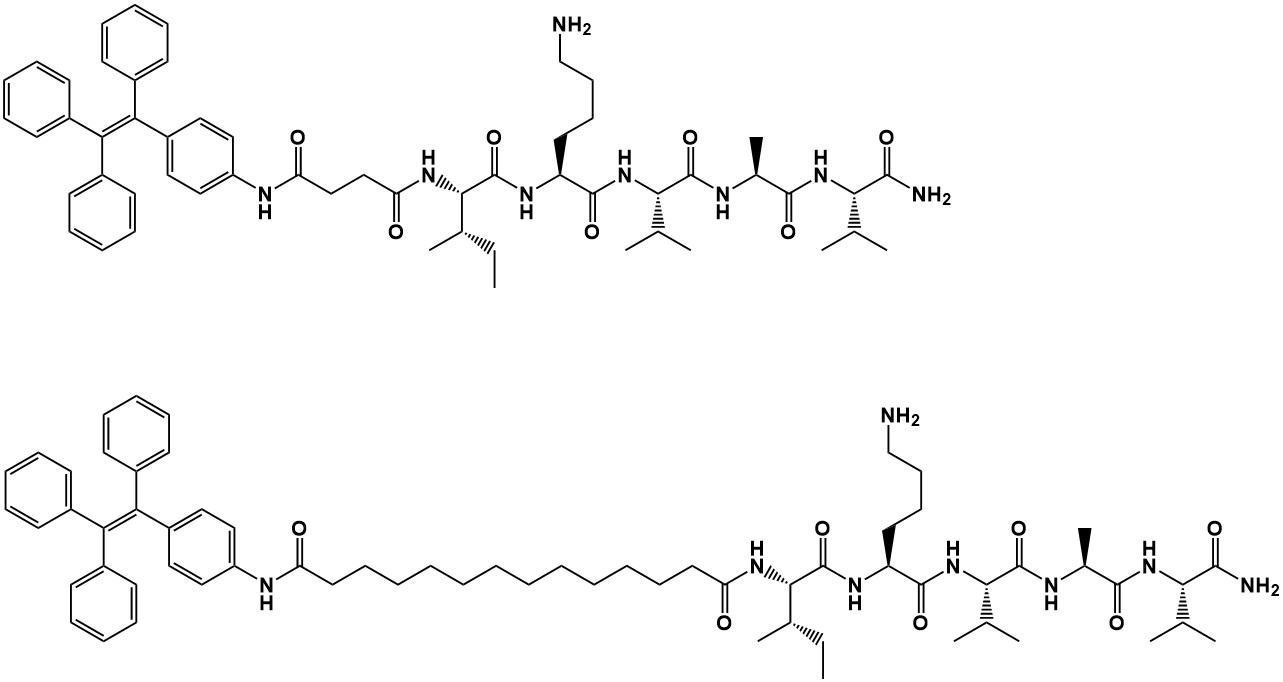
**

**
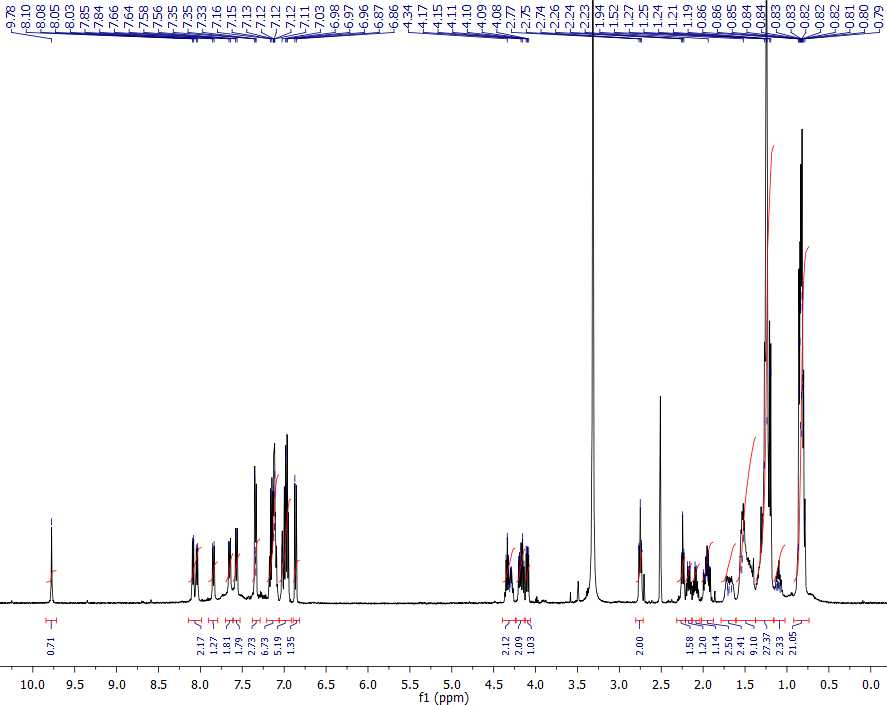
**

**Figure S35**. ^1^H NMR spectrum of compound **5** (500 MHz, DMSO-d_6_).

**
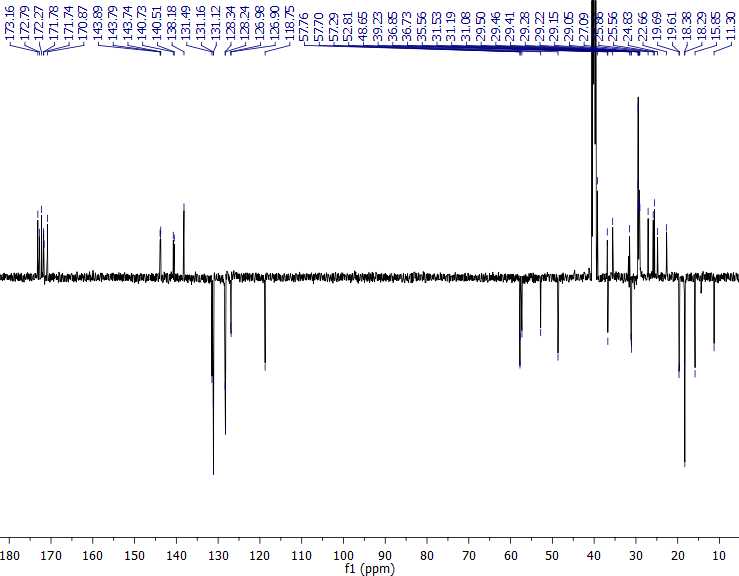
**

**Figure S36**. APT ^13^C NMR spectrum of compound **5** (125 MHz, DMSO-*d_6_*).

**
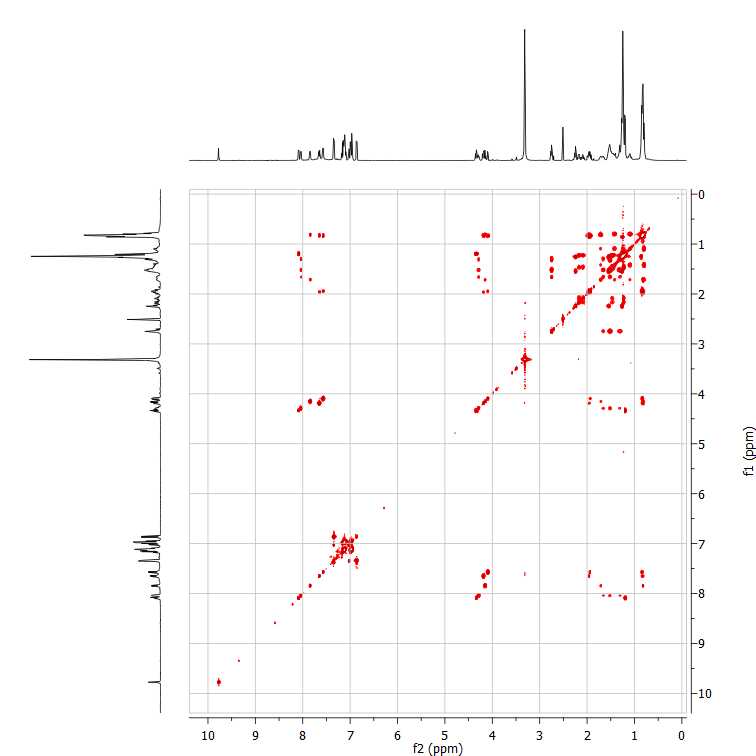

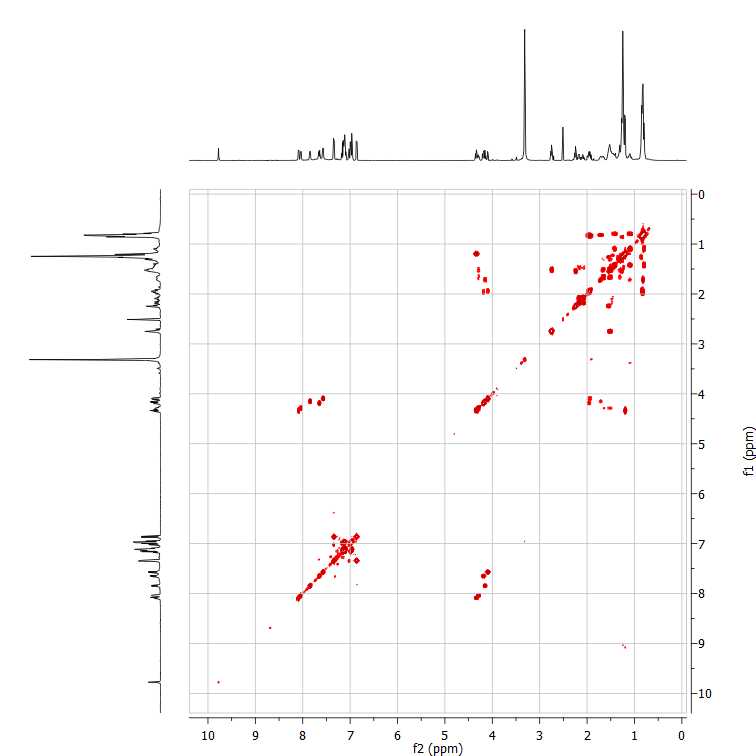
**

**Figure S37.** ^1^H,^1^H**-**COSY spectrum for compound **5** (500 MHz, DMSO-d_6_).

**Figure S38.** ^1^H,^1^H**-**TOCSY spectrum for compound **5** (500 MHz, DMSO-d_6_)**.**

**
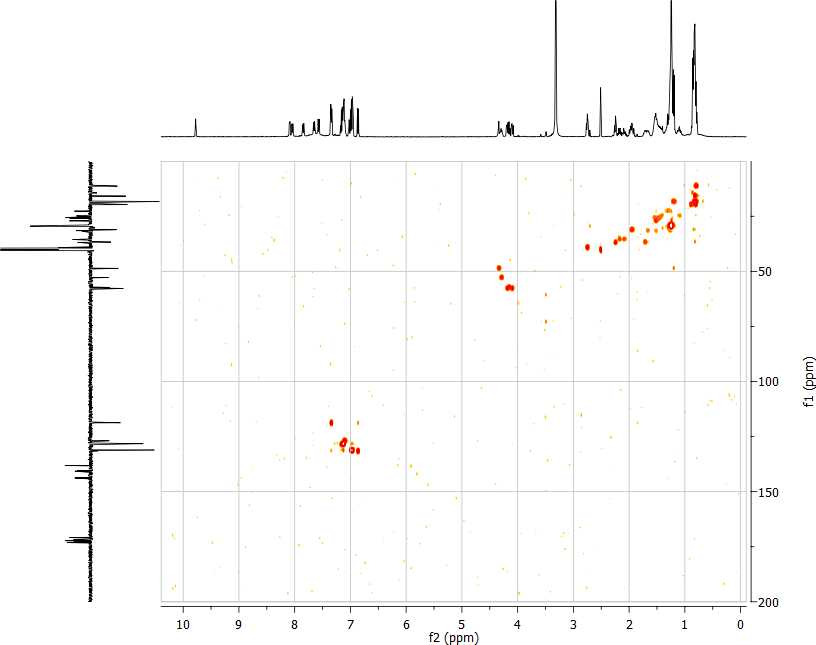

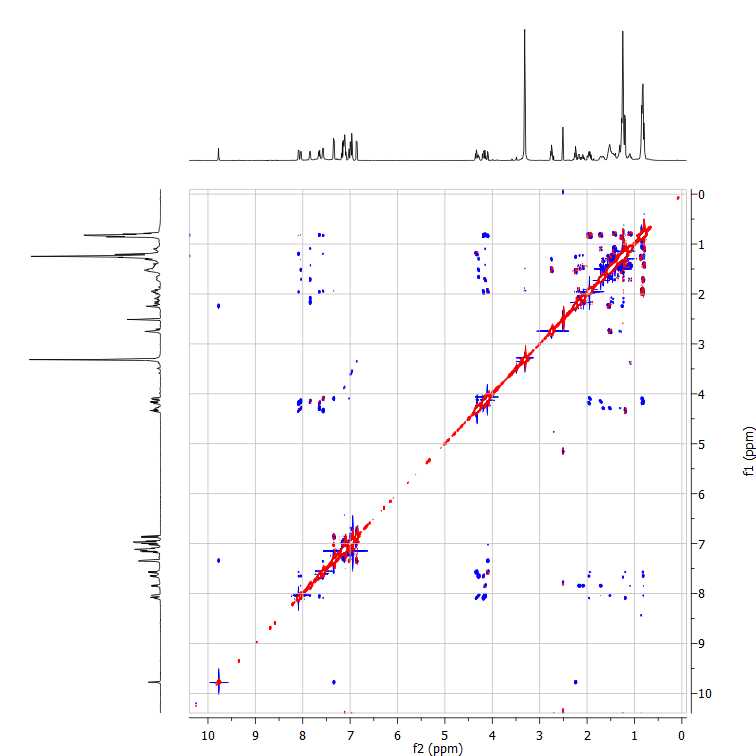
**

**Figure S40**. ^1^H,^13^C**-**HSQC spectrum for compound **5** (500 MHz, 125 MHz, DMSO-d_6_).

**Figure S39.** ^1^H,^1^H**-**ROESY spectrum for compound **5** (500 MHz, DMSO-d_6_, 200 ms).

1. **References**

Kaiser, E. *et al.* (1970) ‘Color test for detection of free terminal amino groups in the solid-phase synthesis of peptides’, *Analytical Biochemistry*, 34(2), pp. 595–598. doi:https://doi.org/10.1016/0003-2697(70)90146-6.

McMurry, J.E. (1989) ‘Carbonyl-coupling reactions using low-valent titanium’, *Chemical Reviews*, 89(7), pp. 1513–1524. doi:10.1021/cr00097a007.
